# Age-Dependent Gut Microbiota Dynamics and Their Association with Male Life-History Traits in *Drosophila melanogaster*

**DOI:** 10.1007/s00248-025-02640-y

**Published:** 2025-11-24

**Authors:** Zahida Sultanova, Handan Melike Dönertaş, Alejandro Hita, Prem Aguilar, Berfin Dag, José Ignacio Lucas-Lledo, Amparo Latorre, Pau Carazo

**Affiliations:** 1 School of Biological Sciences, University of East Anglia, Norwich, UK; 2 Leibniz Institute on Aging – Fritz Lipmann Institute (FLI), Beutenbergstrasse 11, 07745 Jena, Germany; 3 Cluster of Excellence Balance of the Microverse, Friedrich Schiller University Jena, Jena, Germany; 4 Cavanilles Institute of Biodiversity and Evolutionary Biology, University of Valencia, Valencia, Spain; 5 CIBIO Research Centre in Biodiversity and Genetic Resources, InBIO, Universidade Do Porto, Porto, Portugal; 6 BIOPOLIS Program in Genomics, Biodiversity and Land Planning, CIBIO, Campus de Vairão, 4485-661 Vairão, Portugal; 7 Departamento de Biologia, Faculdade de Ciências da Universidade Do Porto, Porto, Portugal; 8 Laboratory of Computational Biology, VIB Center for AI & Computational Biology (VIB.AI), Louvain, Belgium; 9 VIB-KU Leuven Center for Brain & Disease Research, Louvain, Belgium; 10 Department of Human Genetics, KU Leuven, Louvain, Belgium; 11 Institute for Integrative Systems Biology, University of Valencia-CSIC, Valencia, Spain

**Keywords:** Gut microbiota, *Drosophila melanogaster*, Life-history evolution

## Abstract

Growing evidence suggests that the gut microbiota is closely intertwined with life-history evolution in a wide range of species, including well-studied model organisms like *Drosophila melanogaster.* Although recent studies have explored the relationship between gut microbiota and female life-history, the link between gut microbiota and male life-history remains relatively unexplored. In this study, we investigated how gut microbiota changes with male age as well as the associations between gut microbiota composition and male life-history traits in *D. melanogaster*. Using 22 isolines from the *Drosophila melanogaster* Genetic Reference Panel (DGRP), we measured lifespan, early/late-life reproduction, and early/late-life physiological performance. We characterized the gut microbiota composition in young (5 days old) and old (26 days old) flies using 16S rDNA sequencing. We observed substantial variation in both male life-history traits and gut microbiota composition across isolines and age groups. Using machine learning, we show that gut microbiota composition could predict the chronological age of the organisms with high accuracy. The most important species contributing to machine learning prediction belonged to the *Acetobacter* and *Ralstonia* genera. Associations between gut microbiota and life-history traits were also notable, particularly involving different species from the *Acetobacter* genus. Our findings suggest that taxa such as *Acetobacter* may be relevant to the evolutionary ecology of host–microbe interactions in male fruit flies.

**Supplementary Information:**

The online version contains supplementary material available at 10.1007/s00248-025-02640-y.

## Introduction

Understanding the mechanisms of aging and reproduction is crucial to unravel the complex dynamics of life-history evolution [1]. An emerging line of research highlights the role of gut microbiota [2, 3], which significantly influences host lifespan, healthspan, reproduction, and behavior [4–6]. These effects can be mediated by extending metabolic capabilities beyond the host genome and modulating the immune system [7]. Notably, microbiota transfer from young to old individuals can rejuvenate hosts, enhancing both lifespan and late-life reproduction [3, 8–10], but see [11, 12]. Together, these findings position gut microbiota as a central component of life-history evolution [7].

The existing research on life-history evolution has focused predominantly on females, resulting in a pronounced sex bias [13]. This has led to greater emphasis on the connection between gut microbiota and female life-history, while male life-history remains comparatively underexplored. For example, the effects of gut microbiota on female lifespan and reproduction, and to some extent male lifespan, have been well studied in model organisms like *Drosophila melanogaster* [14–17]. In contrast, their role in male reproduction is far less understood [18].

The relatively few studies exploring the influence of gut microbiota on male fitness suggest notable effects on mating behavior and reproductive success [18, 19]. For example, Morimoto et al. (2017) infected *D. melanogaster* males with *Acetobacter pomorum* or *Lactobacillus plantarum*, two of the five most abundant bacterial species in wild flies [20] and known to affect *D. melanogaster* physiology and behavior [21]. Males infected with *L. plantarum* exhibited longer mating durations and caused the females to produce more offspring in the short term, while females mated with males infected with *A. pomorum* were less likely to produce viable offspring. Likewise, Ami et al. (2010) studied how gut microbiota can affect the mating behavior of the Mediterranean fruit fly (*Ceratitis capitata*). First, they disrupted the gut bacterial community of males by sterilizing them with radiation. Then, they found that regenerating the original microbiota community of males, by feeding them with a bacteria-enriched diet, enhanced their mating performance compared to controls [19]. Finally, Heys et al. (2020) found that an intact microbiota is essential for old males to attract females in *D. pseudoobscura* fruit flies [22]. Altogether, these findings show that gut microbiota is an essential factor that affects male fitness.

Our aim in this study was to explore the link between male gut microbiota and life-history by examining gut microbiota composition and male life-history traits across two ages of male *D. melanogaster*. First, we measured key traits in male fruit flies from 22 different DGRP inbred isolines, including life-history traits (lifespan and early/late-life reproduction) and physiological performance traits (early/late-life anti-predatory escape ability). Second, we characterized the early and late life gut microbiota of these isolines and investigated how gut microbiota composition changed with age. Finally, we explored the potential link between these male life-history traits and gut microbiota composition.

## Materials and Methods

### Experimental Population

As focal flies, we used flies from the *D. melanogaster* genetic reference panel (DGRP, see [23]). Hence, individuals within each isoline can be considered clones. Using DGRP isolines (many individuals with the same genotype) instead of wild-type flies allowed us to characterize the life-history traits of different genotypes in standard conditions, while characterizing the early and late life gut microbiota associated with these same genotypes. We fed the DGRP flies with Bloomington Drosophila Stock Centre Cornmeal Food (15.9 g yeast, 9.2 g soy flour, 67.1 g yellow cornmeal, 5.3 g agar, 70 mL light corn syrup, and 4.4 mL propionic acid per Litre). We maintained each isoline inside a bottle with 75 mL of food until the life-history assays. We also maintained sparkling poliert (*spa*) flies to be used as standard competitors (males) and mating partners (females) in the mating assays. We fed *spa* flies with a diet that contains 40 g yeast, 50 g sugar, 10 g soy flour, 60 g corn flour, 10 g agar, 3 g nipagin, and 5 mL propionic acid per Litre. DGRP and *spa* flies were maintained on their standard diets because switching flies to diets that they are not adapted to can strongly affect their behavior and life-history traits [24]. As our main focus was life-history traits and gut microbiota of DGRP flies, the following assays were all performed by using the DGRP diet.

### Life-History Assays

**Fig. 1:**
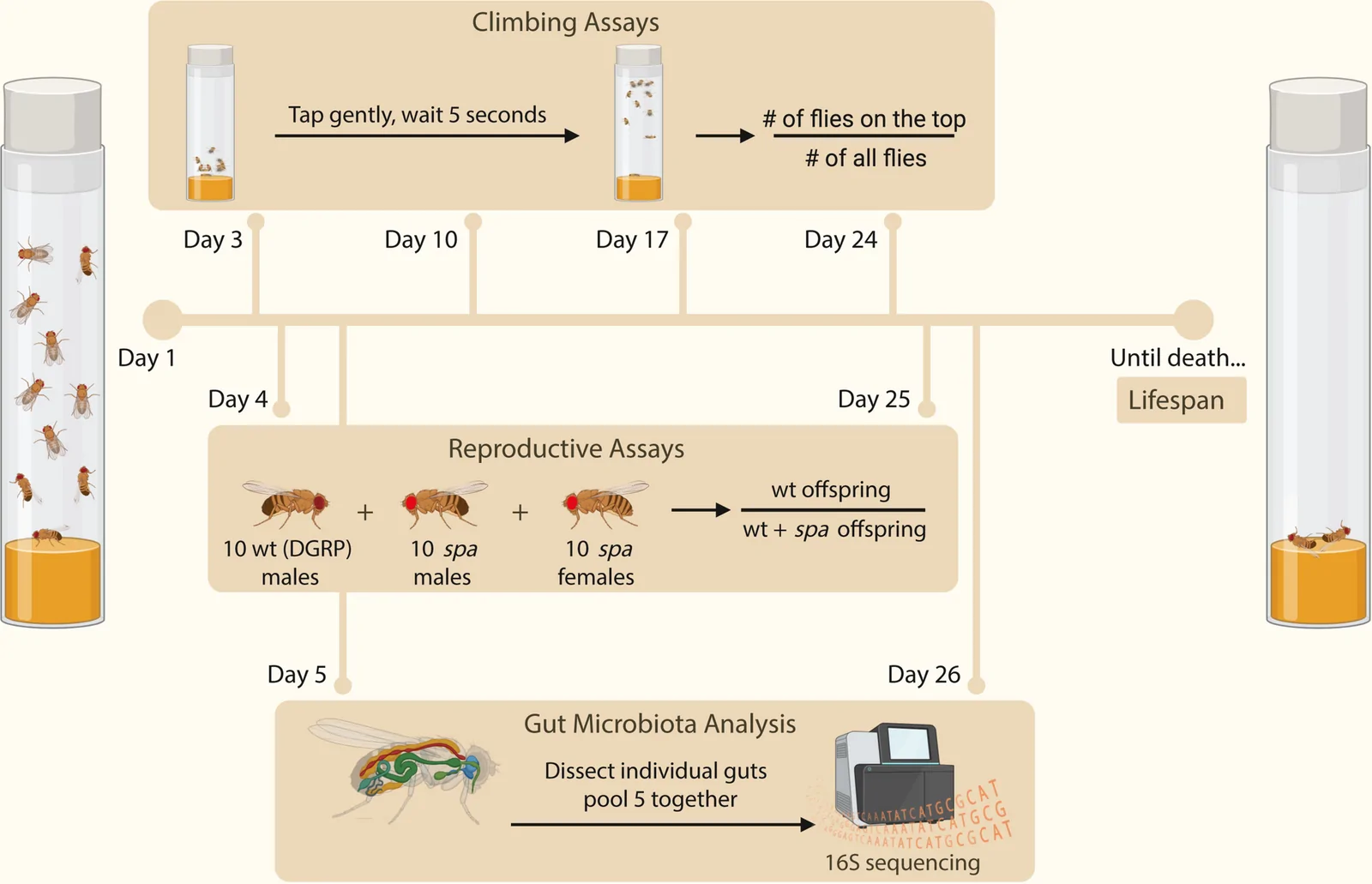
Experimental design. We monitored replicate vials that contained 10 males in same-sex groups for each of the 22 different DGRP isolines. We measured climbing speed for the first four weeks, reproductive success early and late in life (days 4 and 25), and lifespan until they died. We dissected guts right after the mating assays (days 5 and 26). This figure was created with BioRender.com

We set up 9 to 10 replicate vials containing 10 males in same-sex groups for each of the 22 different DGRP isolines that were randomly chosen among the ones without *Wolbachia* infection **(**Table 1**)**. However, we found that isolines 492 and 427 were infected by *Wolbachia* **(**Fig. 3c**)**. It is unclear whether these infections were present before arriving at our laboratory or originated during maintenance in our laboratory. We transferred these flies to new vials with fresh food once a week throughout their lifespan (or until sacrificed, see below), and checked mortality 5–6 days a week by recording the number of dead individuals in each experimental vial. Density within vials was kept constant between 8 and 11 individuals (by re-distributing the flies from the low-density vials among the remaining vials) until the density decreased inevitably later in the experiment as the last flies died **(**Fig. 1**)**.

**Table 1:** Summary of experimental design parameters

Number of isolines	22
Number of vials per isoline	**9–10**
Number of flies per vial	**10**
Replicates for sequencing	**3–4**

To estimate the competitive reproductive success of focal males, we measured the relative paternity of all experimental males competing against standard rivals at two different time points: early (4 days old) and late (25 days old) in life. We used homozygous recessive *spa (sparkling poliert)* mutants as standard mating partners and rivals because their distinctive eye phenotype allows us to distinguish the offspring of wild-type (DGRP) and *spa* parents in competitive fitness assays [25]. For each time point, we introduced 10 focal males with 10 *spa* males and 10 *spa* females into new vials and let them interact and lay eggs for 24 h. At the end of this period, we recovered the focal males belonging to the DGRP isolines and discarded the *spa* individuals. We incubated the eggs and left them to develop into adults for 16 days, froze them, and then counted the relative number of wild-type offspring (number of wild-type offspring divided by the total number of offspring). We calculated reproductive aging by subtracting average late life reproductive success from average early life reproductive success per isoline. Following each reproductive assay, we sacrificed 15–20 males per isoline for gut dissection and kept the remaining flies for life-history characterization.

We also estimated the climbing speed of each isoline once a week for 4 weeks (i.e., on days 3, 10, 17, and 24) by tapping each experimental vial and measuring the proportion of flies that were able to climb to the top in 5 s. We repeated this procedure three times per vial and took the average. Then, we calculated the average across all replicate groups within each isoline as a measure of the climbing speed of each isoline at each time point and estimated functional senescence as the slope of the age-related decline in climbing speed in 4 weeks.

### Gut Dissection, Bacterial DNA Isolation, and Sequencing

For gut dissections, we sacrificed 15–20 flies per isoline right after the two reproduction time points and pooled them in groups of five, resulting in 3–4 replicates per isoline for each age group. We took the flies from two vials per isoline and transferred the remaining unsacrificed flies in the vials to other vials of the isoline to control for density. We dissected each fly separately inside PBS droplets under the microscope by using sterilized forceps. We collected isolated guts in groups of five to have 3–4 biological replicates per isoline and immediately flash-froze them in liquid nitrogen prior to DNA extraction. We performed DNA extraction from gut tissue with the JetFlex ™ Genomic DNA Purification Kit (Genomed, Löhne, Germany). We then quantified DNA with a Nanodrop-1000 Spectrophotometer (Thermo Scientific, Wilmington, DE) and then sent it for sequencing. DNA obtained from gut was used as a template for amplification of the V3–V4 region of bacterial 16S rRNA genes and sequenced in two sequencing runs using the Illumina MiSeq technology at the Foundation for the Promotion of Sanitary and Biomedical Research (FISABIO), Valencia, Spain.

### Processing of the 16S Amplicon Sequencing Data

We used FastQC [26] to assess the quality and length distribution of the reads supplied by the sequencing center and ensured that the sequences did not contain any adapter or primer sequences. The dada2 pipeline, available as an R package [27], was employed to trim, filter, denoise, merge, and remove chimeras. Based on quality profiles, reads were truncated to 280 bp (forward) and 250 bp (reverse), followed by filtering. There were two sequencing runs. The first and second runs retained between 94–99.9% and 94–100% of all reads post-filtering. Errors were estimated independently for the two runs, and subsequently, denoising was performed. Paired-end reads (forward and reverse) were then merged, resulting in abundance tables. On average, 95% of the filtered pairs from run 1 and 92% from run 2 were successfully merged. The abundance tables from both runs were then combined. We employed the “consensus” method to remove chimeric sequences; that is, samples in a sequence table underwent individual processing for chimeric sequences, with a consensus decision made for each sequence variant. Notably, while 70.4% of unique sequences were chimeric, they accounted for 20.7% of the total reads. For taxonomic assignment, we leveraged the dada2 implementation of the RDP naive Bayesian classifier method described in [28] and the Silva taxonomic database (v138) [29]. This enabled the successful assignment of phylum-level information to 85.4% of all ASVs, and species-level information to 49.35% of all ASVs. Subsequently, we employed the DECIPHER R package [30] to construct a multiple alignment. Using the phangorn R package [31, 32], a phylogenetic tree was created based on a Generalized Time Reversible with Gamma rate variation (GTR + G + I) maximum likelihood tree, using a neighbor-joining tree as the starting reference.

For taxonomic assignment, we used the Silva v138.1 database with default confidence thresholds in the dada2 assign Taxonomy function. It should be noted that species-level identification from 16S rRNA amplicon sequencing is inherently limited due to insufficient sequence variation between closely related species, regardless of the database or confidence threshold used. After quality filtering, approximately 75% of retained ASVs received species-level assignments (Supplementary Figure S1i). To address this limitation, we conducted analyses at multiple taxonomic levels (ASV, species, and genus) and explicitly report the number of unidentified ASVs throughout our results.

#### ASV Filtering

Prior to ASV level filtering, we identified 2020 ASVs. We employed four sequential criteria for the quality filtering of ASVs: (1) We used only ASVs with lengths ranging from 400 to 465 bp, resulting in 1672 ASVs. (2) We selected ASVs with phylum-level taxonomic assignment, narrowing it down to 1593 ASVs. (3) We excluded ASVs that were phylogenetically distant from all other ASVs, leaving 1523 ASVs. (4) We excluded taxa that are present in less than 1% of all samples, leaving 685 ASVs only. The ASV length distribution, taxonomic assignment rate, and phylogenetic tree both before and after ASV filtering can be viewed in Supplementary Figure S1. We further explored whether any DNA extraction or sequencing batches correlated with the presence of ASVs that were filtered out. Some DNA extraction batches contained a higher amount of these ASVs (Supplementary Figure S2). This suggests that these ASVs could indeed be technical artifacts.

#### Sample Filtering

We observed significant variations in library sizes across the samples. Four samples with fewer than 10,000 reads were excluded from the analysis (Supplementary Figure S3). We retained only those isolines for which we had at least three replicate samples at both the early- and late-life time points, yielding 22 isolines with a total of 161 samples. Given the pronounced disparities in library size, we standardized the number of reads by rarefaction to 10,000 reads for each sample to enable a more equitable comparison of diversity and abundance. While we present the results using only one such rarefied table (unless indicated otherwise), to ensure our results were not skewed by a single non-representative rarefaction, we generated 100 distinct rarefied tables and conducted our analysis on each.

### Analysis of 16S Amplicon Sequencing Data

#### Alpha and Beta Diversity

We employed the “estimate_richness” function from the phyloseq package [33] to compute alpha diversity and the distance function for beta diversity calculations. Our analysis incorporated five alpha diversity metrics: “Observed” (number of observed taxa) and three metrics accounting for both richness and evenness: “Shannon”, “Simpson,” and “InvSimpson” (Inverse Simpson). For beta diversity, we used the Bray–Curtis distance. The number of taxa across samples and the prevalence of each taxon (i.e., the number of samples in which each taxon occurs) were determined based on the abundance matrix and non-zero occurrences.

#### PCA and PCoA on Microbial Abundances

We conducted a PCA on CLR-transformed abundance values using the prcomp function from base R and the transform function within the microbiome R package [34]. Additionally, PCoA plots were generated using the ordinate function from the phyloseq R package [33], based on the Bray–Curtis distance matrix derived from log10(count + 1) transformed data.

### Age and Life-History Prediction Using Microbial Abundances

We used “caret” R package [35] to train a random forest model to predict age class (i.e., “early” vs. “late”) or life-history traits (i.e., early climbing speed, late climbing speed, functional aging, early reproductive success, reproductive aging, lifespan) using species-level CLR transformed abundance data (number of features = 27). This choice balanced biological interpretability with the available taxonomic resolution, acknowledging that approximately 25% of ASVs lacked species-level annotation. We used 5 times repeated fivefold cross-validation to train the model. Importantly, train and test data included a non-overlapping set of isolines, preventing data leakage. This design ensures that the model is always tested on completely novel genetic backgrounds, providing more stringent validation against overfitting than approaches like permutation testing, which would test within the same set of isolines. The predicted age categories, thus, represent chronological rather than physiological stages, as isolines varied substantially in their lifespans (see Results). While biological age normalized by isoline lifespan could provide additional insights, our experimental design prioritized preventing data leakage by ensuring train and test sets contained non-overlapping isolines, precluding the use of isoline-specific lifespan information in predictive models. We trained the model on 15 isolines and their 111 samples and tested it on 7 isolines, and their 50 samples. Accuracy was calculated using “confusionMatrix” function for age prediction. For performance evaluation, we used accuracy and no information rate (NIR) for age classification, where NIR represents the baseline accuracy achieved by always predicting the majority class. For regression models predicting life-history traits, we used R^2^ (coefficient of determination) to assess variance explained and MAE (Mean Absolute Error) to quantify prediction errors, comparing both against their respective no information baselines. Models were considered successful if they substantially outperformed these baselines. All performance metrics for the 20 independent model iterations are reported in Supplementary Tables 1 and 2. We used the “varImp” function from the “caret” package to identify the most important features (i.e., species).

Because our primary goal was to evaluate whether microbiota can predict age or life-history traits, not to deploy a predictive model, we sought to ensure that the small sample size and random train/test splits did not undermine our conclusions. Consequently, we repeated the entire procedure 20 times with new train–test splits, resulting in 20 different models. We reported the performance range of all models in the results section and all results are given as Supplementary Tables 1  and 2.

### Age and Isoline Effects on Gut Microbiota Composition

We used DESeq2 R package [36] to analyze differential ASV abundances between early- and late-life and between isolines. We used the ASV abundances rarefied to 10,000 reads per sample and analyzed only the ASVs that are found in at least 5 samples in early- and late-life and have on average 1 in 10,000 abundance across early- and late-life samples. We used a model that takes run (the sequencing run, 2 levels); DNA batch (DNA extraction batch); age (early vs. late); and isoline (DGRP isoline, 22 levels) into account and we tested for the significance of age and isoline separately, using a likelihood ratio test. Specifically, we fitted the following DESeq2 model: “ASV abundance ~ run + DNAbatch + age + Isoline.” We used likelihood ratio tests to assess the significance of age and isoline effects. For testing age effects, we compared the full model against a reduced model excluding age (~ run + DNAbatch + Isoline). For testing isoline effects, we compared the full model against a reduced model excluding isoline (~ run + DNAbatch + age). This approach accounts for technical variation from sequencing runs and DNA extraction batches while testing for biological effects of interest. ASVs with FDR-adjusted-*p*-value < 0.1 are considered significant.

### Linking Life-History Traits to Gut Microbiota Composition

We collected all ASVs that show significant differences between isolines, aggregated ASV level information to species level information, and then tested for association between their abundances and life-history traits, using the Spearman correlation test. We chose Spearman correlation for its robustness to non-normal distributions common in microbiome data and its ability to detect monotonic relationships without assuming linearity, which is appropriate for our proportional and rate-based traits. We calculated the mean abundance level of each bacterial species for each isoline and age category and used these values to correlate with life-history. *p*-values are corrected using the FDR procedure as implemented in “p.adjust” function in base R and an FDR-adjusted *p*-value < 0.1 is considered as a significant association.

## Results

Using DGRP isolines, we explored the association between various life-history traits and gut microbiota. Following the quality filtering of microbiota samples (see Methods), we analyzed 22 isolines. We investigated five key traits related to life-history and physiological performance: average lifespan, early climbing speed, functional aging, early reproductive success, and reproductive senescence **(**Fig. 1**)**.

**Fig. 2:**
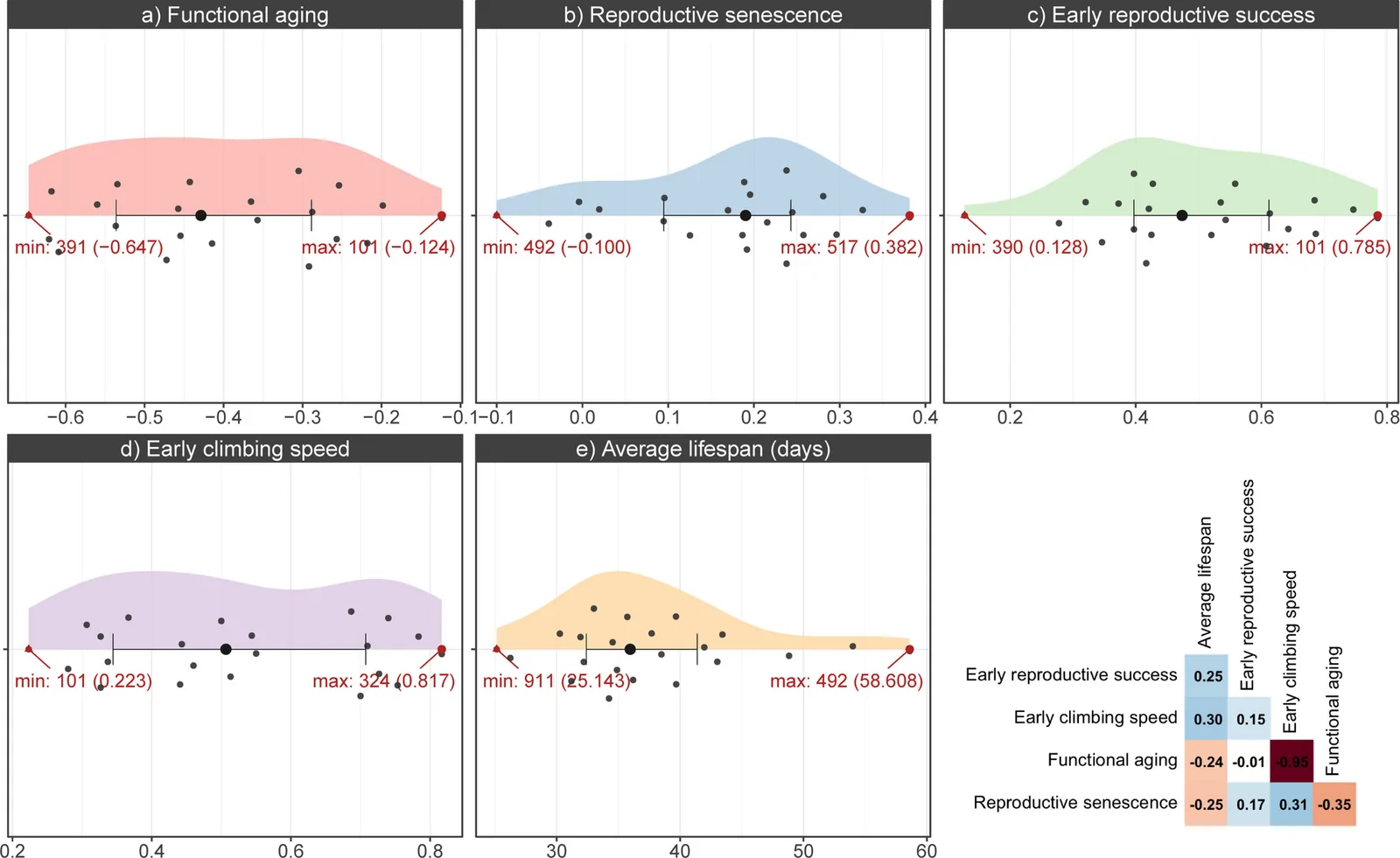
Variation in life-history traits across DGRP isolines. Distributions of life-history traits: functional aging (slope of the decline in climbing ability with age) (**a**), reproductive senescence (decline in reproductive success with age) (**b**), early reproductive success (**c**), early climbing speed (**d**), and average lifespan (days) (**e**) are shown as half-violin plots across the 22 isolines. Median and IQR are annotated with a point and error bar, respectively. Isolines with maximum and minimum values are annotated on each plot. **f** Pairwise correlations (Spearman’s *ρ*) among the five traits, with color intensity indicating correlation strength and sign

We noted considerable variation in life-history traits across isolines. For instance, the longest recorded lifespan was 2.3 times the shortest lifespan. Similarly, the highest early climbing speed was 3.6 times the lowest value, and the highest recorded early reproductive success was 6.16 times the lowest. The mean lifespan stood at 37.8 days with a standard deviation of 8.23 days **(**Fig. 2, see Supplementary Tables 3 and 4 for more details). We further analyzed the correlations between the life-history traits and did not observe significant correlations except for early climbing speed and functional aging (Fig. 2f,Supplementary Figure S4).

### Diversity of Gut Microbiota Across DGRP Lines in Early and Late Life

**Fig. 3:**
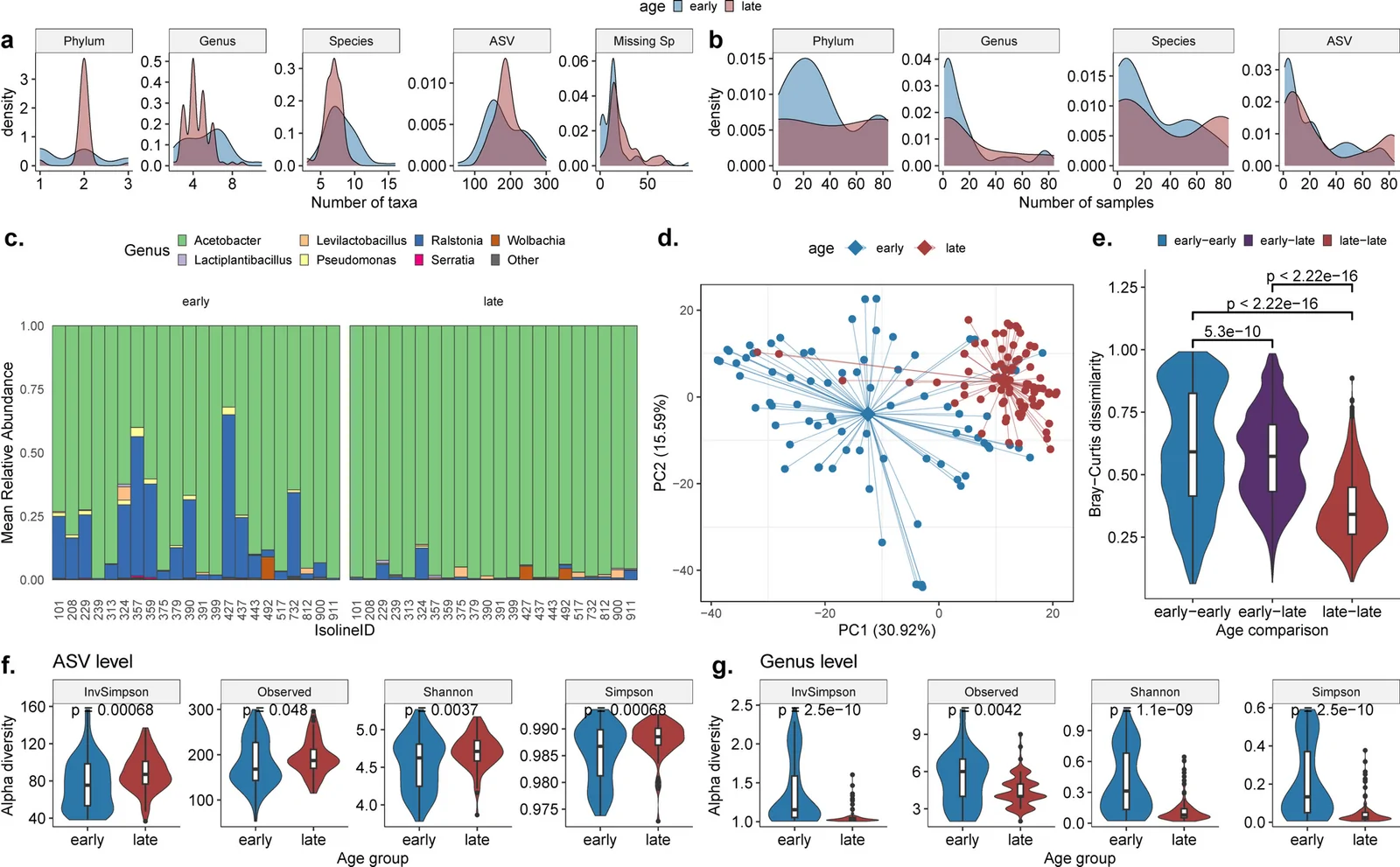
Early and late life microbiota across DGRP lines. **a** Density plots displaying the diversity of taxa across early and late-life samples for various taxonomic ranks. “Missing Sp” indicates the number of ASVs lacking species-level annotations. **b** Density plots illustrating taxa prevalence in young (blue) and old (red) samples across distinct taxonomic levels. **c** Mean relative abundance of genera across isolines, categorized by age. **d** PCA, employing the Euclidean distance on the CLR-transformed abundance matrix (i.e., Aitchison index). Age group centroids are represented by diamonds, and the distances to all samples within the same group are shown by lines originating from the center. **e** Distribution of beta-diversity between samples from different age groups, calculated at the ASV level. ASV-level (**f**) and genus-level (**g**) alpha diversity across age groups, evaluated using various indices. *p*-values shown on the charts correspond to the Wilcoxon test

After quality filtering for Amplicon Sequence Variants (ASV) and samples and performing read rarefaction (see Methods), we identified 677 ASVs across 161 samples. Initially, we analyzed the distribution of detected taxa across samples, at different taxonomic levels **(**Fig. 3a**)**. Samples had a median of 184 ASVs, ranging between 53 and 300. From this pool, on average 20 ASVs (median, 14; range, 0–80) remained unidentified at the species level. Given the inherent limitations of species-level identification from 16S sequencing, we present our results at multiple taxonomic levels. The median counts for recognized species, genera, and phyla were 7 (range, 3–16); 5 (range, 2–11); and 2 (range, 1–3), respectively. These findings align with the literature emphasizing that *Drosophila* gut microbiota mainly comprises a select array of bacterial species, typically between 5 and 20 [37]. We also observed a prominent variation across isolines, with the most diverse isoline hosting twice as many taxa as the least diverse one at nearly all taxonomic tiers (Supplementary Table 5).

Further, we examined the prevalence of taxa at each taxonomic level. ASVs were present in an average of 44.5 out of 161 samples, species in 48, genera in 30.4, and phyla in 75.5 samples **(**Fig. 3b**)**. Notably, even at this general level, we observed differences between young and old samples. Older samples exhibited slightly reduced species and genera diversity, whereas ASV diversity increased **(**Fig. 3a**)**. Distribution of taxa prevalence also varied between the young and old groups, with an increased number of species and ASVs with higher prevalence in older samples **(**Fig. 3b**)**. We further analyzed the taxa prevalence across isolines and found that while many taxa were specific to certain isolines (shared in 1 to 5 isolines), some were consistently found across a large number of isolines, especially at the species and ASV levels (Supplementary Figure S5).

Next, we explored the genera composition across isolines and age groups **(**Fig. 3c**)**, noting a decline in diversity and relative abundance of certain taxa, such as *Ralstonia*, with age. Interestingly, *Wolbachia* was only detected in two isolines: one (isoline 427) only in old flies, and one (isoline 492) both in young and old groups. PCA on CLR transformed data highlighted age-centric clustering (predominantly in the first principal component), which explained 30.92% of the data variance **(**Fig. 3d**)**. Similarly, PCoA conducted on log-transformed abundance data showed age-based clustering, independent of sequencing batches (Supplementary Figure S6).

We then analyzed alpha diversity differences between young and old samples at both ASV and genera levels **(**Fig. 3f–g**)**. Our analysis validated the observed contrasting diversity trends between genera (decreasing with age) and ASV (increasing with age) levels in Fig. 3a. It is pertinent to mention that our samples were pooled, not derived from individual flies, signifying the alpha diversity here represents community-level diversity. Lastly, we examined the beta diversity between young and old samples, utilizing the Bray–Curtis dissimilarity measure **(**Fig. 3e**)**. A marked difference emerged between young and old samples, with older samples showing more similarity amongst themselves than the younger samples.

### Prediction Models

**Fig. 4:**
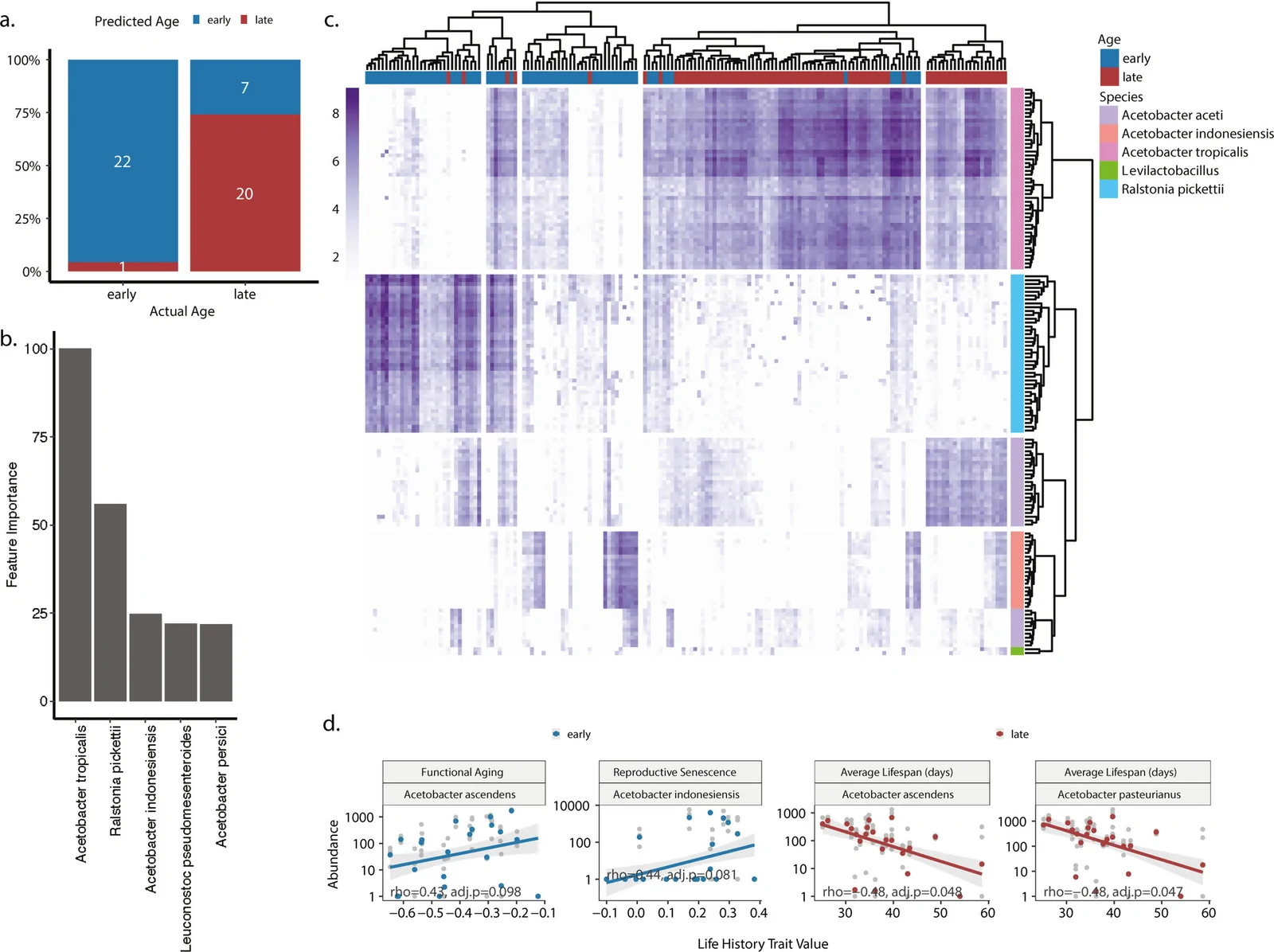
Male age- and life-history-associated taxa in *Drosophila* gut. **a** Confusion matrix showing age-prediction performance using CLR transformed species abundances and a random forest model. Numbers indicate the count of samples: 22 young samples correctly predicted as “early,” 1 young sample incorrectly predicted as “late,” 7 old samples incorrectly predicted as “early,” and 20 old samples correctly predicted as “late.” The overall accuracy is (22 + 20)/(22 + 1 + 7 + 20) = 84%, compared to the no information rate of 54% (27/50, which would be achieved by always predicting the majority class “late”). **b** Most important features in random forest model for prediction of age. **c** ASVs showing differential abundance with age and their species annotations. Color intensity shows log2 transformed count values after pseudocount addition (i.e., count + 1). **d** Significant correlations (FDR-adjusted *p*-value < 0.1) between male life-history traits and abundance values of species that show significant differences between isolines (FDR-adjusted *p*-value < 0.1). Gray points indicate individual samples; colored points represent the mean value for each isoline

Given the large variation in gut microbiota and life-histories, next, we asked whether we could predict age or life-history traits using gut microbiota. We first trained a random forest model on 15 isolines to predict chronological age (early vs. late) using CLR-transformed species level abundance information. We tested the model on the remaining non-overlapping 7 isolines and achieved 84% accuracy (42/50 samples), as the model correctly classified 22 of 23 early-life and 20 of 27 late-life samples **(**Fig. 4a**)**. However, the no information rate, which reflects the accuracy one would get by always predicting the most frequent class, was 54%. The most important species contributing to the classification were *Acetobacter tropicalis*, *Ralstonia pickettii*, *Acetobacter indonesiensis*, *Leuconostoc pseudomesenteroides*, and *Acetobacter persici* **(**Fig. 4b**)**. Then, we trained models to predict life-history traits using microbial abundances. Complete model performance metrics are provided in the supplementary materials (accuracy values for age prediction models in Supplementary Table 1; R^2^ and MAE values for life-history trait regression models in Supplementary Table 2). For age prediction, we achieved a median accuracy of 86.27% (range, 72.92–96%) across 20 independent train-test splits, substantially exceeding the no information rate baseline of 54% (Supplementary Table 1). This indicates robust predictive ability for chronological age. In contrast, life-history trait predictions showed poor performance, with most models achieving R^2^ values below 0.1 and MAE values similar to or worse than the no information baseline (Supplementary Table 2). Moreover, *Acetobacter tropicalis* and *Ralstonia pickettii* were consistently the two most important features across all models, while the remaining three were on average half of the time among the top 5 most important species. We conclude that gut microbiota can predict the chronological age of samples with high accuracy, but not the life-history traits of the isolines, which may reflect limited power given the sample sizes per trait and multiple comparisons.

### Age-Related Changes in Gut Microbiota and Life-History Traits

We found a significant effect of male age, isoline, and their interaction on bacterial abundances. Although there was an overall increase in ASV diversity with male age, the direction of age-specific change in bacterial diversity differed with respect to different isolines. In general, the relative abundance of *Acetobacter* genus increased with age, while other genera such as *Ralstonia* became less abundant **(**Fig. 4c**)**.

When we explored the link between life-history traits and bacterial diversity, we observed patterns that mostly agreed between age groups, while early reproductive success showed divergent patterns. Though none reached statistical significance after correcting for multiple testing (FDR-adjusted *p* > 0.1 for all comparisons; Supplementary Figure S7), the observed number of taxa showed a negative correlation with early reproductive success in early life samples (*ρ* = − 0.31) but a positive correlation in late life samples (*ρ* = 0.23). We interpret these patterns cautiously given their lack of statistical significance (Supplementary Figure S7).

We observed significant associations (FDR adjusted *p*-value < 0.1) between abundances of different *Acetobacter* species and life-history traits. In early life, *A. ascendens* abundance was positively correlated with functional aging and *A. indonesiensis* abundance was positively correlated with reproductive senescence. Finally, late-life abundances of *A. ascendens* and *A. pasteurianus* were both negatively correlated with average lifespan **(**Fig. 4d**)**.

### Testing the Potential Influence of Rarefaction on Results

Since we observed a large variation across library sizes, we rarefied all samples to 10,000 reads before all analyses. Since random sampling of 10,000 reads may bias the results, we then performed rarefaction 100 times and made all calculations. We first checked the alpha diversity differences between early- and late-life samples and confirmed that the trend is observed across all rarefactions (Supplementary Figure S8). Then we checked differential abundances, following the exact same procedure described above, repeated for 100 independent rarefactions. The 5 species reported in the main text that have differential abundance between early- and late-samples were detected as differentially abundant in all 100 rarefactions (Supplementary Table 6). There were two more species *Pseudomonas yamanorum* and *A. persici* that were significant in 30 and 9 of the rarefactions, respectively, but with only 1 or 2 ASVs. We repeated the same analysis for the associations between species and life-history traits (Supplementary Table 7). Three of the four associations reported in Fig. 4d were also detected in the same direction in all 100 rarefactions, while the association between *A. ascendens* in early life and functional aging was observed only in 57 of the associations. A negative association between *P. yamanorum* and reproductive senescence was also observed in both early- and late samples but only in a limited number of rarefactions, 7 and 1 out of 100 rarefactions, respectively.

## Discussion

In this study, we explored the link between gut microbiota and male life-history traits. First, as previously shown in the literature, we found age-related changes in male gut microbiota diversity and composition, with species and genera diversity decreasing with age and *Acetobacter* becoming dominant in the guts of older individuals, in line with the literature in males [20]. Second, we showed that gut microbiota composition could robustly predict the chronological age of male flies while failing to predict their life-history traits. Third, we identified significant associations between gut microbiota composition and several male life-history traits. In particular, the abundance of different species of *Acetobacter* was negatively correlated with lifespan and positively correlated with reproductive and functional aging, suggesting a potential negative impact of these species on male aging and reproduction in fruit flies. These findings suggest that there are consistent age-related shifts in gut microbiota across genotypes; however, microbiota-life-history trait relationship is more complex, requiring experimental manipulation of gut microbiota composition to fully understand potential causal links.

### Age-Related Changes in Male Gut Microbiota

We found that ASV-level bacterial diversity was higher, while species and genus diversity was lower, in older male flies. In humans, studies present mixed findings on how alpha diversity changes with age, and differences in genetic background, culture, and lifestyle (e.g., diet) are suggested as potential reasons for the inconsistencies [38]. In male fruit flies, previous findings show that as flies age OTU diversity is higher [11] while species diversity is lower [20], aligning with our results. The consistency between different fruit fly experiments is probably due to the controlled experimental conditions and diets used for rearing flies, in contrast to human studies.

The observed increase in ASV-level richness alongside a decline in species-level richness may reflect a rise in unidentified ASVs at an old age. However, it is difficult to determine whether this pattern results from limited database annotations, possible within-host evolution (despite the short 21-day timespan), or other technical factors. 

We found that the relative abundance of *Acetobacter* was higher in older individuals, in line with some previous findings [20] but not others [11], underlining that age-related changes in microbiota compositions can differ between populations and isolines. This was accompanied by a decline in the abundance of other species, such as those from the genus *Ralstonia,* with age. Although *Ralstonia* has been identified previously in the fruit fly gut in relatively small amounts [39], its higher abundance in young individuals in our study suggests it may play an important role in some fly strains, including the DGRP lines.

It is important to mention that we pooled 5 genetically identical flies per DGRP isoline to approximate alpha diversity, the microbial diversity within a single sample [40]. In ecological terms, alpha diversity reflects how many different taxa coexist locally and how evenly they are distributed, offering insights into community complexity and resilience. Higher alpha diversity is often associated with ecological stability and functional redundancy, which may buffer the host against environmental or physiological stressors—an especially relevant consideration in the context of aging, where declining resilience is a common theme [41]. As we used genetically identical flies reared together, their microbiota can be treated as a single community, though stochastic variation may cause slight differences. Thus, our measure also reflects gamma diversity, the total diversity across multiple individuals or habitats [42]. Gamma diversity in this context provides a snapshot of the overall microbial repertoire within a genetically homogeneous group, and how it may change over time with host aging.

We also examined beta diversity, which reflects variation in microbial community composition between samples, and found that older flies had more similar gut microbiota to each other than younger ones. In ecological terms, beta diversity reflects the extent of differentiation between communities and is often shaped by dispersal, selection, and drift. A decline in beta diversity may suggest a convergence toward a limited set of taxa, possibly reflecting ecological filtering or reduced niche space in the aging gut [43]. This pattern contrasts with findings from human studies, where beta diversity typically increases with age [44, 45]. One possible explanation for this unexpected pattern is technical. Since our samples were pooled from multiple individuals, the reduced variability may reflect a regression toward the mean. Biologically, the lower inter-individual variability in older flies might also result from their rearing under highly controlled conditions with identical diets throughout aging—conditions that differ markedly from the more variable environments experienced by aging humans [38], or wild flies in nature. Alternatively, this may reflect a convergence toward a simplified community dominated by a few taxa, such as *Acetobacter*, in late life. This microbial streamlining could represent a loss of ecological complexity and reduced functional flexibility, potentially contributing to age-associated physiological decline.

Part of the variation in DGRP gut microbiota may come from horizontal transfer during co-housing with *spa* flies. Since DGRP flies were kept on the same diet, variation between isolines should mainly reflect host effects [46]; however, *spa* flies, previously reared on a different diet, could have contributed microbes via feces on the shared food [47]. As *spa* density and ratios were kept constant across assays, their contribution should have been comparable across vials, though some variability may remain. Importantly, both young and old DGRP males experienced the same standardized *spa* exposure, making age-related effects unlikely.

Finally, we were able to predict the chronological age of samples (young vs. old) from gut microbiota composition. Because isolines highly vary in lifespan, 5 and 26 days likely represent different physiological stages across lines (e.g., mid-life vs. late-life). We focused on chronological age to avoid data leakage in machine learning, since incorporating biological age would require isoline-specific lifespan data not available for novel lines. Future studies with larger panels could address this by grouping isolines with similar lifespans. Despite this limitation, our results show that microbiome composition reliably predicts chronological age across diverse genotypes, pointing to robust, clock-like changes in the gut microbiome.

### Linking Gut Microbiota with Male Life-History Traits

We found no correlation between bacterial diversity and male life-history traits. Previous studies in humans have generally linked greater microbial diversity to better health [48]. However, the literature has mixed findings regarding the impact of bacterial diversity on male life-history in flies, with most research focusing on lifespan rather than reproduction. While a more diverse microbiome has been linked to increased lifespan in both sexes [49], experiments with axenic flies showed that introducing multiple bacterial species progressively reduced lifespan, with axenic flies living the longest and those inoculated with five species having the shortest lifespans [17]. The absence of a link between bacterial diversity and male life-history traits in our study could be due to our limited statistical power, given the relatively small sample size (i.e., 22 isolines). Alternatively, it is possible that bacterial diversity does not significantly influence male life-history in fruit flies. In fact, a recent study found that feeding old male fruit flies with the gut microbiome of young flies affects neither locomotor activity nor lifespan [11], suggesting the limited ability of gut microbiome to modulate male life-history in this species. Further studies with larger sample sizes are needed to explore these possibilities.

Although bacterial diversity was not associated with male life-history, we did find specific links between the abundance of certain bacterial species and aging-related traits. In particular, certain species of *Acetobacter* (i.e., *A. ascendens*, *A. indonesiensis*, and *A. pasteurianus)* were linked to faster actuarial, functional or reproductive aging. These findings contrast with Wesseltoft et al. (2024) that showed transferring young microbiota into old males had no effect on lifespan or locomotor activity. Several factors can explain this difference. First, despite the presence of beneficial bacterial species in young guts, the aged guts of old flies may already be dysfunctional, rejecting the young microbiota and limiting its effect on life-history (i.e., host-mediated effects, see [50]). Second, transplantation of beneficial microbiota might be more effective earlier in life, before aging-related damage has already accumulated [10]. Finally, it is noteworthy that the observed correlations between certain bacterial species and life-history traits in our study indicate associations rather than causation. Additional research is needed to determine the functional roles of these species, their interactions with the host, and the underlying mechanisms through which they may influence lifespan.

Our findings linking late-life *A. indonesiensis* abundance to reproductive senescence highlight the potential negative effects of this species on male reproduction. A previous study demonstrated that males mono-associated with *A. pomorum* had shorter mating duration and lower number of offspring compared to those mono-associated with *L. plantarum* [18]. While *Acetobacter* species are generally linked with benefits to female reproduction [17, 51, 52], our findings, together with those of Morimoto et al. (2017), suggest that certain species of *Acetobacter* may have an adverse effect on male reproduction. Further research is required to investigate how mono or poly-associations with different bacterial species affect male reproductive success, ideally in more competitive environments.

Finally, we also found correlations between the abundance of *A. ascendens* and *A. pasteurianus* with ageing, specifically faster functional ageing and shorter lifespan. A previous correlational study by Walters et al. (2020) also linked *Acetobacter* abundance to faster ageing [53]. They observed that female flies from low-latitude populations had shorter lifespans, higher early reproduction, and more acetic acid bacteria (including *Acetobacter*), while the ones from high-latitude populations exhibited longer lifespans, lower early reproduction, and fewer acetic acid bacteria. Likewise, Gould et al. (2018) found that bacterial combinations that caused short lifespan in both sexes led to high fecundity, and combinations that caused long lifespan resulted in a low fecundity. These findings suggest that gut microbiota composition can drive a life-history trade-off between lifespan and reproduction in females. However, since male reproductive success was not investigated in these studies, it remains unclear whether a similar trade-off exists in males. Given that we found no positive associations between *Acetobacter* species and male fitness traits, it is also possible that certain gut microbiota compositions promote high fecundity and short lifespan in females while conferring no benefit to males, potentially due to sexually antagonistic selection [54].

## Conclusion

Our study reveals that gut microbiota composition in male *D. melanogaster* undergoes significant age-related changes, with *Acetobacter* species becoming increasingly dominant in late life and correlating with shorter lifespan, faster functional decline, and reproductive aging. While microbial diversity patterns shifted across taxonomic levels with age, we found no consistent associations between overall diversity and male life-history traits. Notably, gut microbiota composition accurately predicted chronological age but failed to predict life-history traits, suggesting that, while age-associated microbial shifts are robust, the current experimental setup lacks the resolution or power to capture microbiota-life-history links. These findings underscore the need for future mechanistic and longitudinal studies to uncover the causal roles of specific microbial taxa in shaping male aging and reproductive strategies.

## Supplementary Information

Below is the link to the electronic supplementary material.

Supplementary Figure S1 (PDF. 8.56 MB)

Supplementary Figure S2 (PDF. 9.83 KB)

Supplementary Figure S3 (PDF.4.69 KB)

Supplementary Figure S4 (PDF.352 KB)

Supplementary Figure S5 (PDF.5.29 KB)

Supplementary Figure S6 (PDF. 63.4 KB)

Supplementary Figure S7 (PDF. 740 KB)

Supplementary Figure S8 (PDF. 6.35 KB)

Supplementary Figure Legends (DOCX. 7.32 KB)

Supplementary Tables (XLSX.37.0 KB)

## Data Availability

Raw sequencing data have been deposited in the European Nucleotide Archive (ENA) at EMBL-EBI under accession number PRJEB88786. Life-history trait data is available in Supplementary Table 3. All processed data, including the data underlying each figure, is available in BioStudies with accession number S-BSST2029.

## Code availability

All code for preprocessing, statistical analysis, and visualization is available at https://github.com/mdonertas/DGRP_16S_MaleLH. The repository includes complete R scripts for all analyses, an renv.lock file for full reproducibility, and detailed documentation of all computational methods used in this study.
