# Supplementary material for: Age-Dependent Gut Microbiota Dynamics and Their Association with Male Life-History Traits in Drosophila melanogaster
Source: Microb Ecol. 2025 Nov 24;89:5. doi: 10.1007/s00248-025-02640-y (PMC12743032; doi:10.1007/s00248-025-02640-y)
Supplement: Supplementary file 1 [file 248_2025_2640_MOESM1_ESM.pdf]

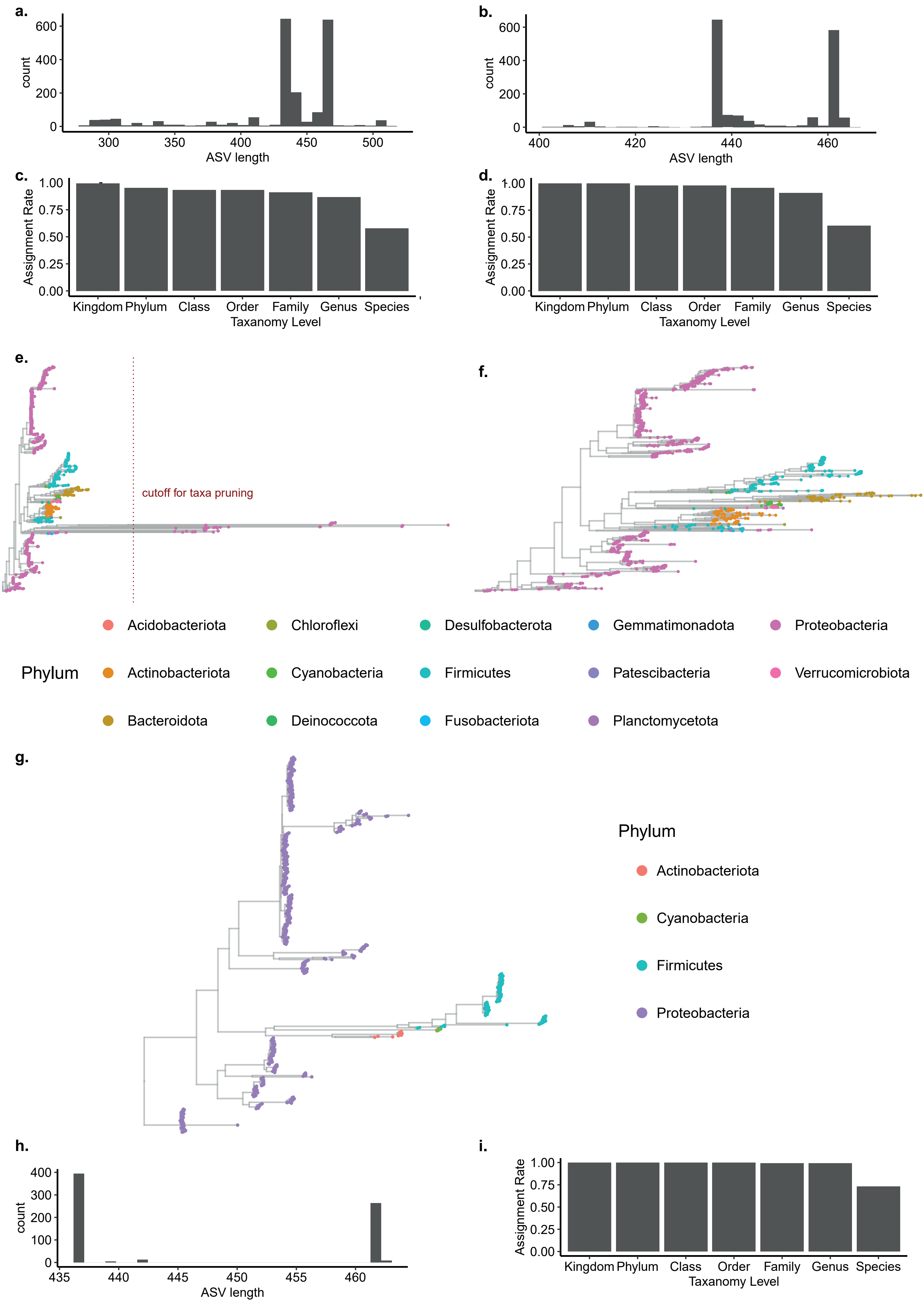
a.
count
ASV length
b.
count
ASV length
c.
Assignment Rate
Kingdom Phylum Class Order Family Genus Species
Taxanomy Level
d.
Assignment Rate
Kingdom Phylum Class Order Family Genus Species
Taxanomy Level
e.
cutoff for taxa pruning
f.
Phylum
Acidobacteriota
Actinobacteriota
Bacteroidota
Chloroflexi
Cyanobacteria
Deinococcota
Desulfobacterota
Firmicutes
Fusobacteriota
Gemmatimonadota
Patescibacteria
Planctomycetota
Proteobacteria
Verrucomicrobiota
g.
Phylum
Actinobacteriota
Cyanobacteria
Firmicutes
Proteobacteria
h.
count
ASV length
i.
Assignment Rate
Kingdom Phylum Class Order Family Genus Species
Taxanomy Level
