# Supplementary material for: Age-Dependent Gut Microbiota Dynamics and Their Association with Male Life-History Traits in Drosophila melanogaster
Source: Microb Ecol. 2025 Nov 24;89:5. doi: 10.1007/s00248-025-02640-y (PMC12743032; doi:10.1007/s00248-025-02640-y)
Supplement: Supplementary file 2 [file 248_2025_2640_MOESM2_ESM.pdf]

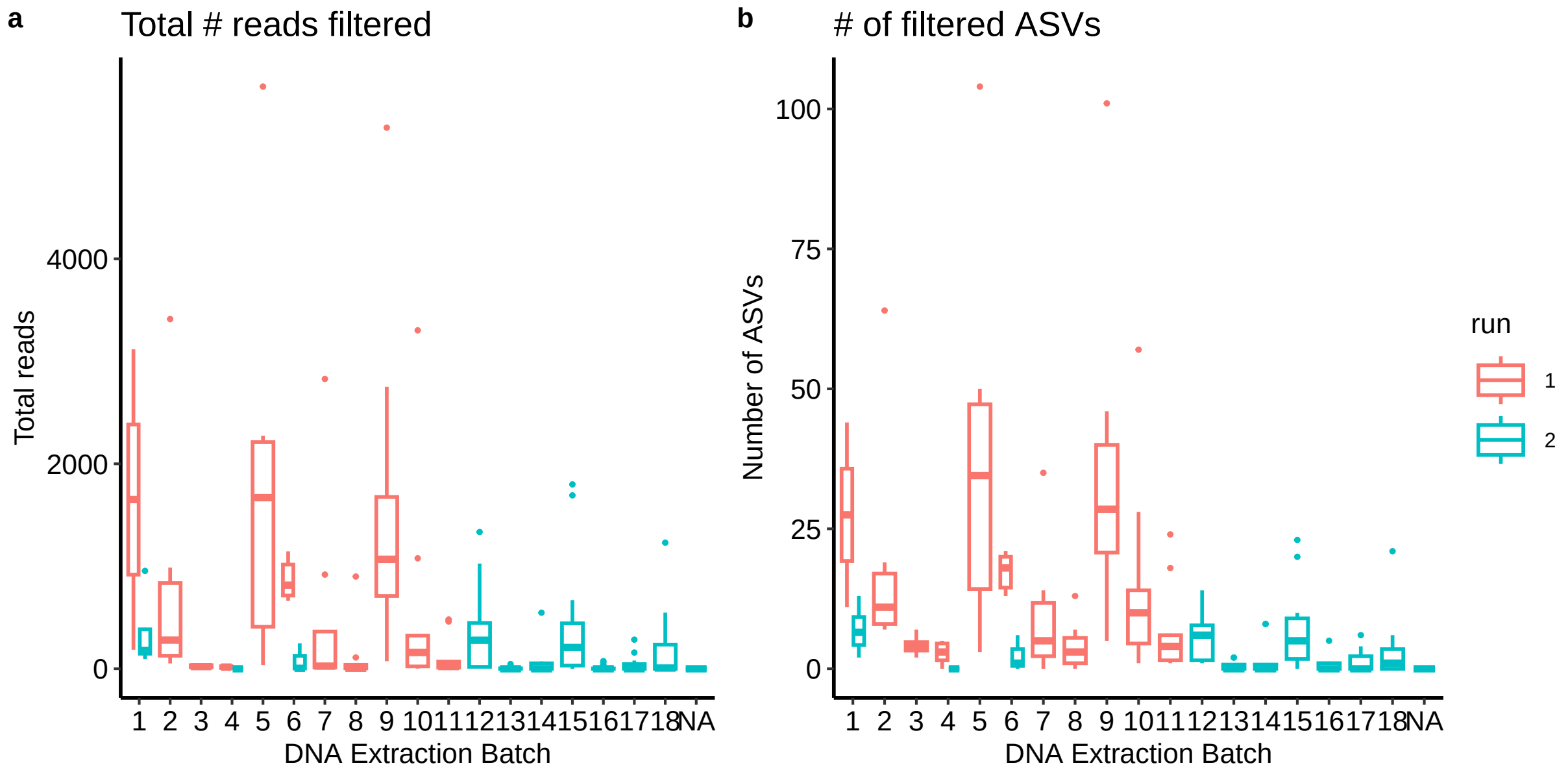
a
Total # reads filtered
Total reads
4000
2000
0
1 2 3 4 5 6 7 8 9 10 11 12 13 14 15 16 17 18 NA
DNA Extraction Batch
b
# of filtered ASVs
Number of ASVs
100
75
50
25
0
1 2 3 4 5 6 7 8 9 10 11 12 13 14 15 16 17 18 NA
DNA Extraction Batch
run
1
2
