# Supplementary material for: Age-Dependent Gut Microbiota Dynamics and Their Association with Male Life-History Traits in Drosophila melanogaster
Source: Microb Ecol. 2025 Nov 24;89:5. doi: 10.1007/s00248-025-02640-y (PMC12743032; doi:10.1007/s00248-025-02640-y)
Supplement: Supplementary file 3 [file 248_2025_2640_MOESM3_ESM.pdf]

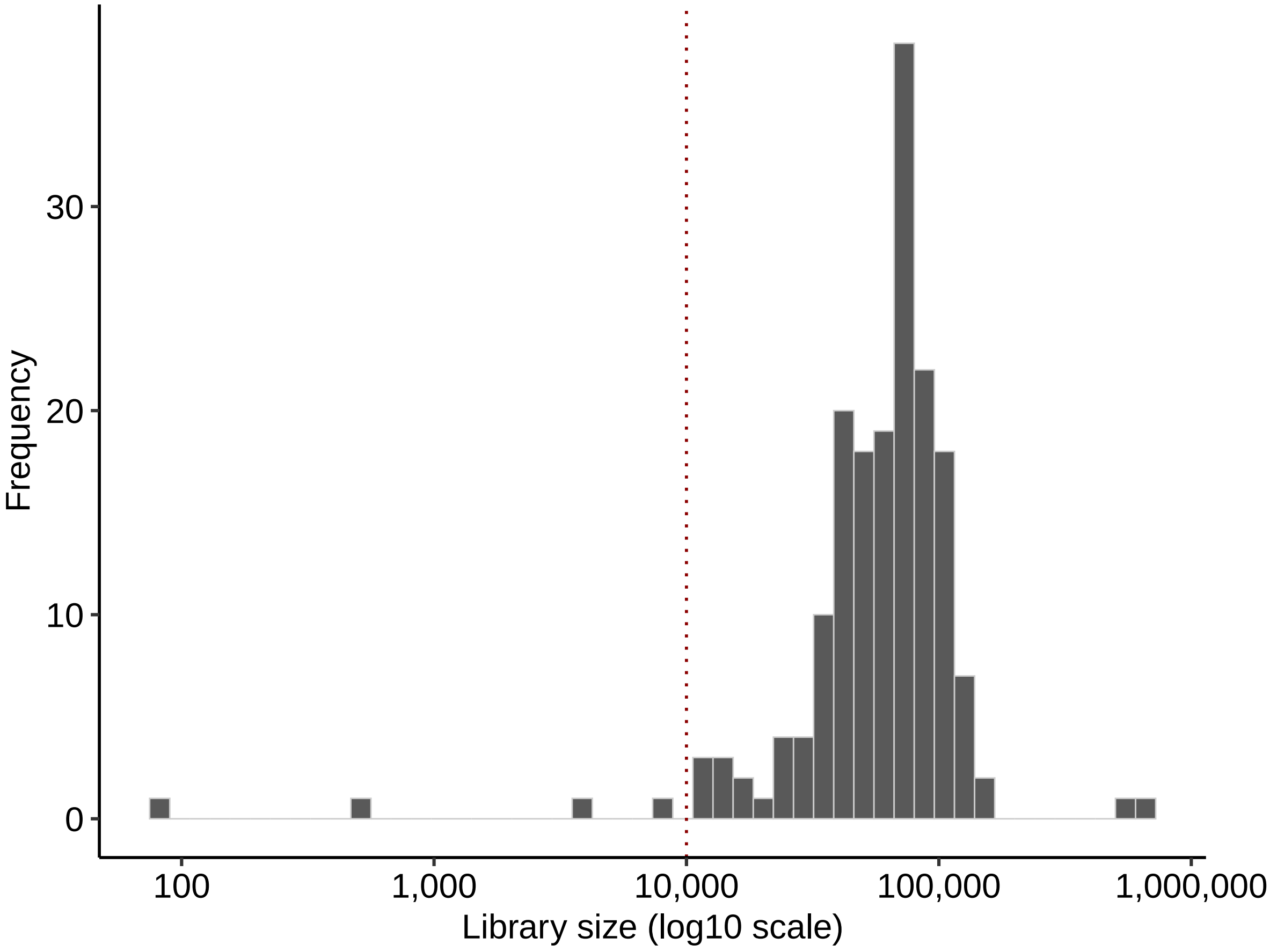

Frequency
30
20
10
0
100
1,000
10,000
100,000
1,000,000
Library size (log10 scale)
