# Supplementary material for: Age-Dependent Gut Microbiota Dynamics and Their Association with Male Life-History Traits in Drosophila melanogaster
Source: Microb Ecol. 2025 Nov 24;89:5. doi: 10.1007/s00248-025-02640-y (PMC12743032; doi:10.1007/s00248-025-02640-y)
Supplement: Supplementary file 4 [file 248_2025_2640_MOESM4_ESM.pdf]

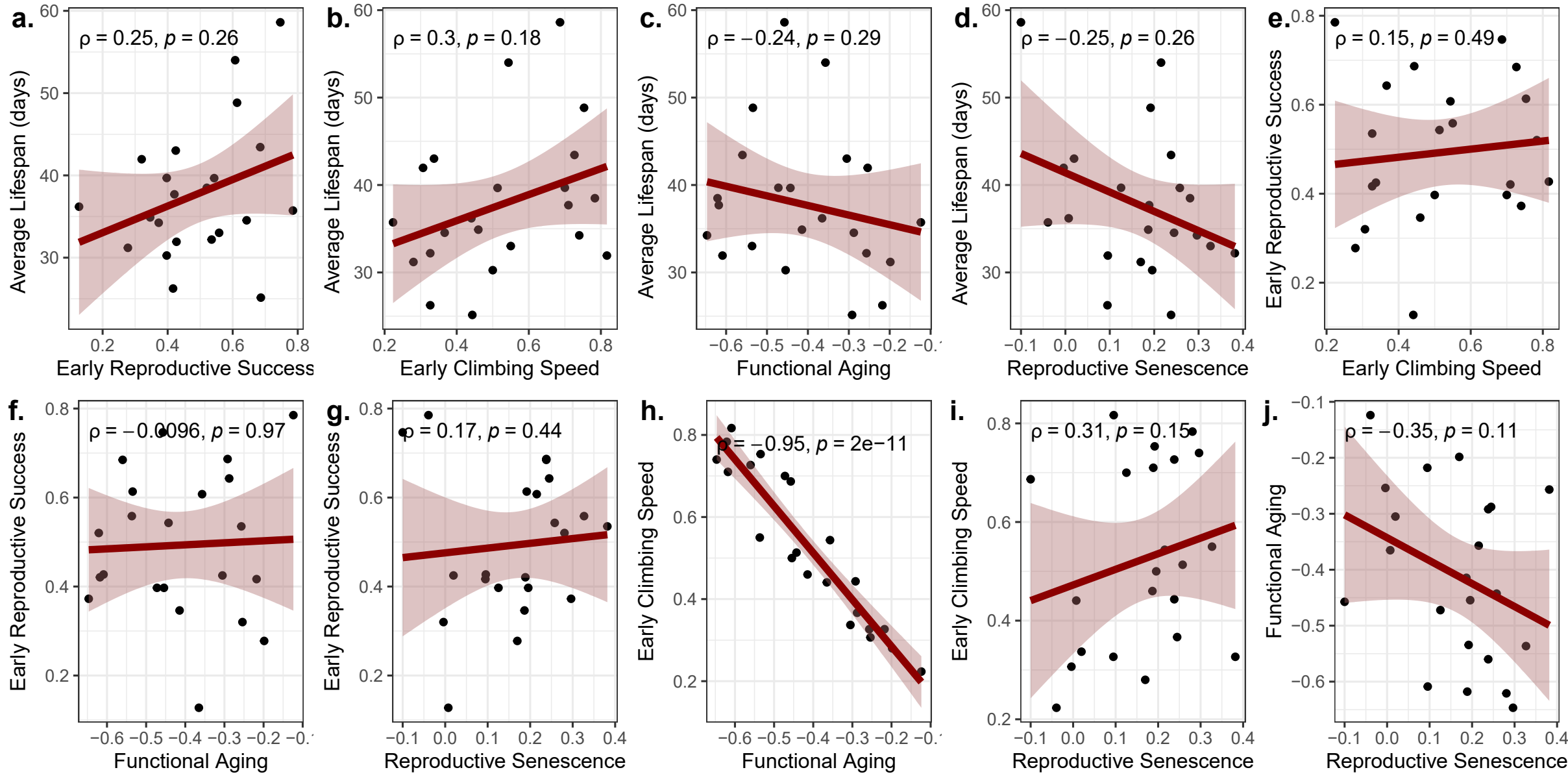

a.
ρ = 0.25, p = 0.26
Average Lifespan (days)
Early Reproductive Success
b.
ρ = 0.3, p = 0.18
Average Lifespan (days)
Early Climbing Speed
c.
ρ = −0.24, p = 0.29
Average Lifespan (days)
Functional Aging
d.
ρ = −0.25, p = 0.26
Average Lifespan (days)
Reproductive Senescence
e.
ρ = 0.15, p = 0.49
Early Reproductive Success
Early Climbing Speed
f.
ρ = −0.0096, p = 0.97
Early Reproductive Success
Functional Aging
g.
ρ = 0.17, p = 0.44
Early Reproductive Success
Reproductive Senescence
h.
ρ = −0.95, p = 2e−11
Early Climbing Speed
Functional Aging
i.
ρ = 0.31, p = 0.15
Early Climbing Speed
Reproductive Senescence
j.
ρ = −0.35, p = 0.11
Functional Aging
Reproductive Senescence
