# Supplementary material for: Age-Dependent Gut Microbiota Dynamics and Their Association with Male Life-History Traits in Drosophila melanogaster
Source: Microb Ecol. 2025 Nov 24;89:5. doi: 10.1007/s00248-025-02640-y (PMC12743032; doi:10.1007/s00248-025-02640-y)
Supplement: Supplementary file 6 [file 248_2025_2640_MOESM6_ESM.pdf]

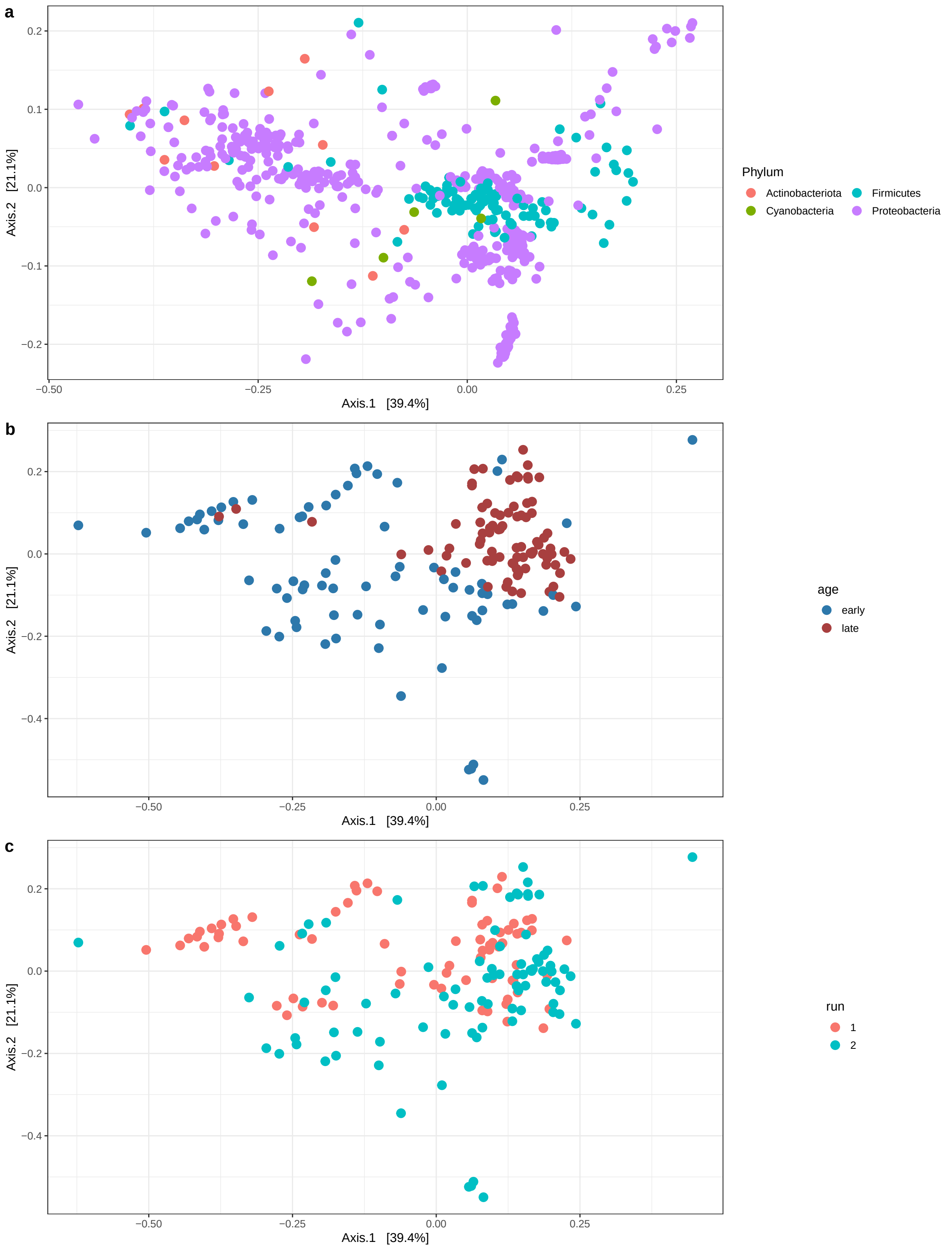

a
0.2
0.1
0.0
−0.1
−0.2
Axis.2 [21.1%]
−0.50
−0.25
0.00
0.25
Axis.1 [39.4%]
Phylum
Actinobacteriota
Firmicutes
Cyanobacteria
Proteobacteria
b
0.2
0.0
−0.2
−0.4
Axis.2 [21.1%]
−0.50
−0.25
0.00
0.25
Axis.1 [39.4%]
age
early
late
c
0.2
0.0
−0.2
−0.4
Axis.2 [21.1%]
−0.50
−0.25
0.00
0.25
Axis.1 [39.4%]
run
1
2
