# Supplementary material for: Age-Dependent Gut Microbiota Dynamics and Their Association with Male Life-History Traits in Drosophila melanogaster
Source: Microb Ecol. 2025 Nov 24;89:5. doi: 10.1007/s00248-025-02640-y (PMC12743032; doi:10.1007/s00248-025-02640-y)
Supplement: Supplementary file 7 [file 248_2025_2640_MOESM7_ESM.pdf]

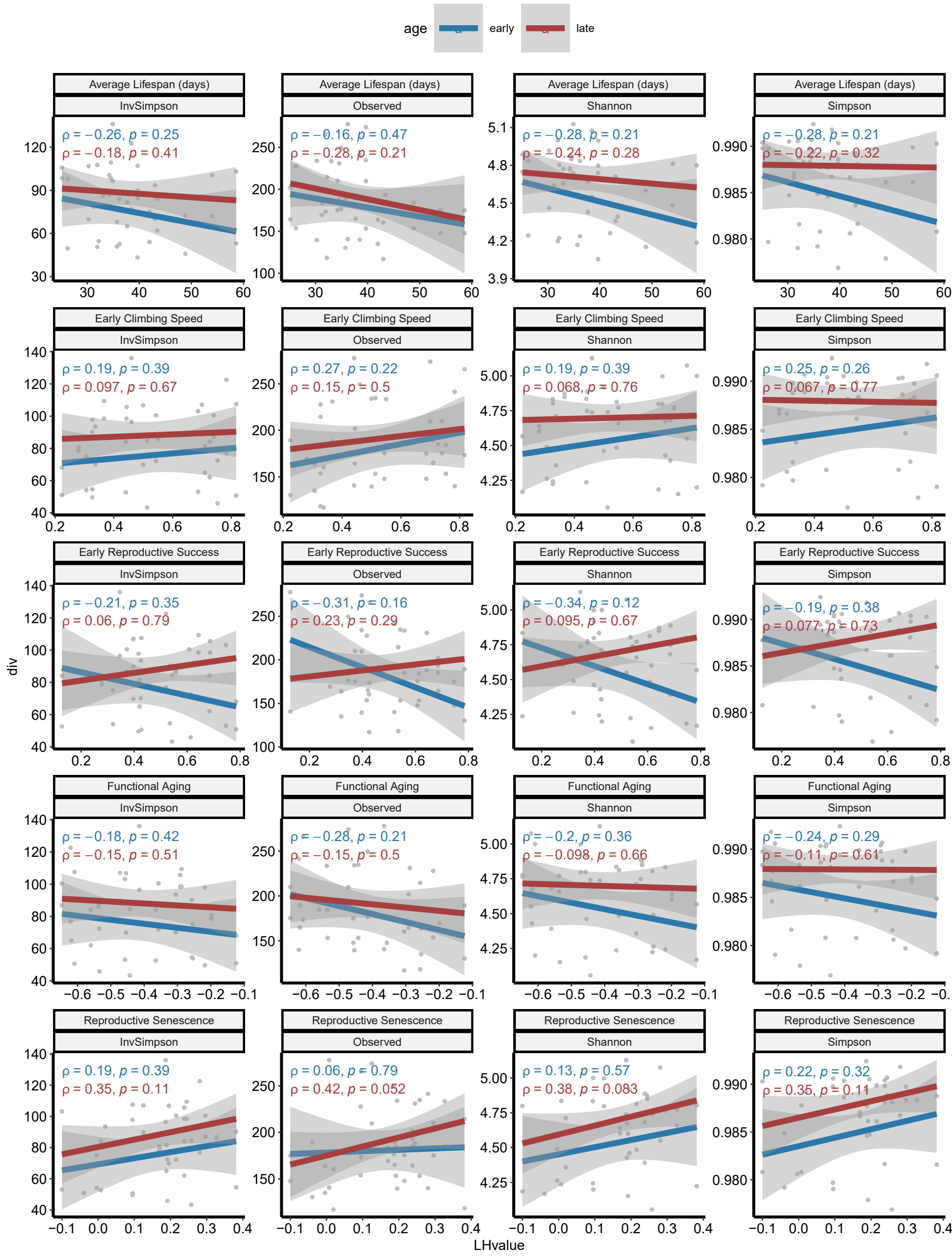
age
early
late
Average Lifespan (days)
InvSimpson
ρ = −0.26, p = 0.25
ρ = −0.18, p = 0.41
Average Lifespan (days)
Observed
ρ = −0.16, p = 0.47
ρ = −0.28, p = 0.21
Average Lifespan (days)
Shannon
ρ = −0.28, p = 0.21
ρ = −0.24, p = 0.28
Average Lifespan (days)
Simpson
ρ = −0.28, p = 0.21
ρ = −0.22, p = 0.32
Early Climbing Speed
InvSimpson
ρ = 0.19, p = 0.39
ρ = 0.097, p = 0.67
Early Climbing Speed
Observed
ρ = 0.27, p = 0.22
ρ = 0.15, p = 0.5
Early Climbing Speed
Shannon
ρ = 0.19, p = 0.39
ρ = 0.068, p = 0.76
Early Climbing Speed
Simpson
ρ = 0.25, p = 0.26
ρ = 0.067, p = 0.77
Early Reproductive Success
InvSimpson
ρ = −0.21, p = 0.35
ρ = 0.06, p = 0.79
Early Reproductive Success
Observed
ρ = −0.31, p = 0.16
ρ = 0.23, p = 0.29
Early Reproductive Success
Shannon
ρ = −0.34, p = 0.12
ρ = 0.095, p = 0.67
Early Reproductive Success
Simpson
ρ = −0.19, p = 0.38
ρ = 0.077, p = 0.73
Functional Aging
InvSimpson
ρ = −0.18, p = 0.42
ρ = −0.15, p = 0.51
Functional Aging
Observed
ρ = −0.28, p = 0.21
ρ = −0.15, p = 0.5
Functional Aging
Shannon
ρ = −0.2, p = 0.36
ρ = −0.098, p = 0.66
Functional Aging
Simpson
ρ = −0.24, p = 0.29
ρ = −0.11, p = 0.61
Reproductive Senescence
InvSimpson
ρ = 0.19, p = 0.39
ρ = 0.35, p = 0.11
Reproductive Senescence
Observed
ρ = 0.06, p = 0.79
ρ = 0.42, p = 0.052
Reproductive Senescence
Shannon
ρ = 0.13, p = 0.57
ρ = 0.38, p = 0.083
Reproductive Senescence
Simpson
ρ = 0.22, p = 0.32
ρ = 0.35, p = 0.11
div
LHvalue
