# Supplementary material for: Age-Dependent Gut Microbiota Dynamics and Their Association with Male Life-History Traits in Drosophila melanogaster
Source: Microb Ecol. 2025 Nov 24;89:5. doi: 10.1007/s00248-025-02640-y (PMC12743032; doi:10.1007/s00248-025-02640-y)
Supplement: Supplementary file 8 [file 248_2025_2640_MOESM8_ESM.pdf]

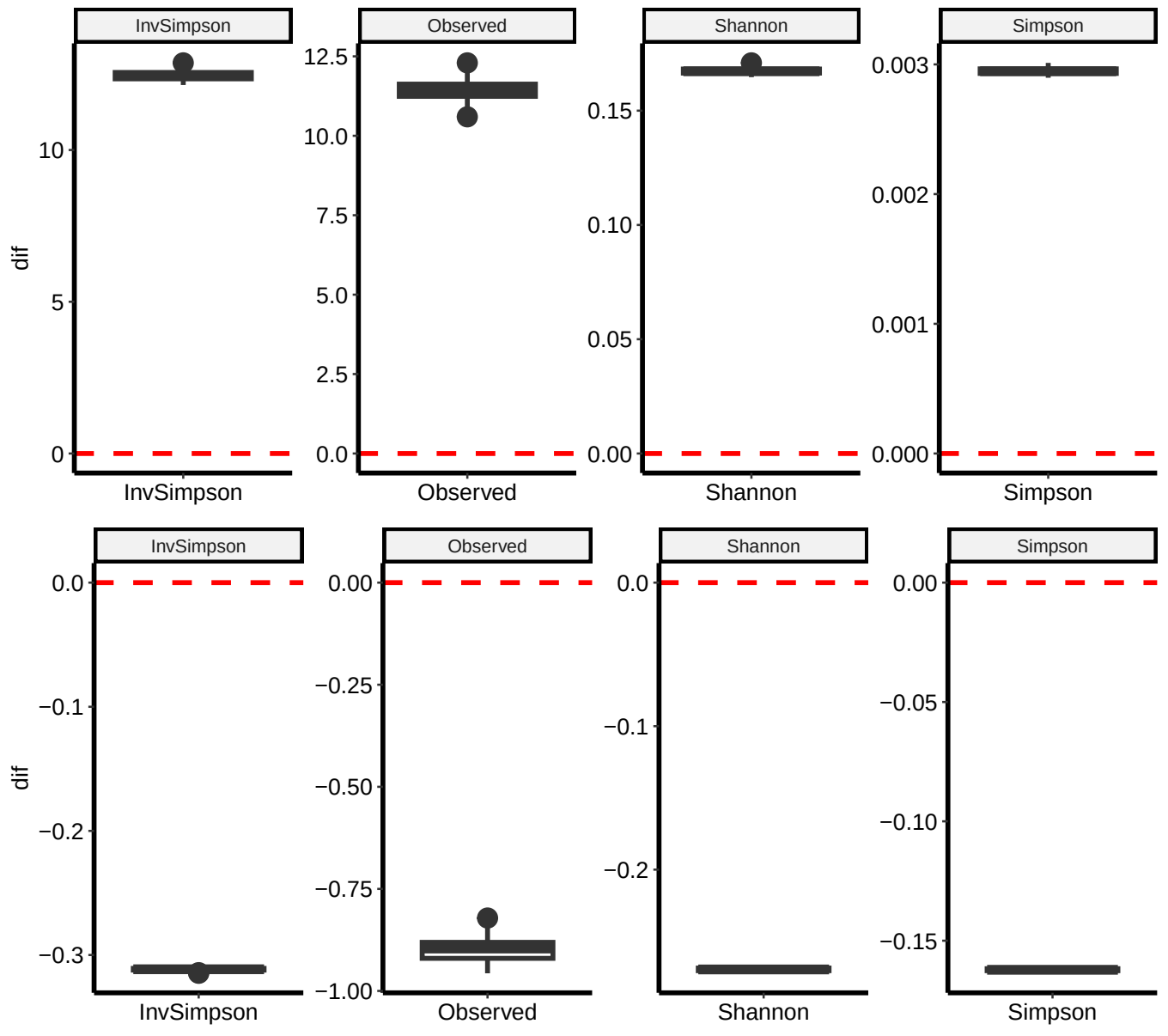

InvSimpson
Observed
Shannon
Simpson
dif
10
5
0
12.5
10.0
7.5
5.0
2.5
0.0
0.15
0.10
0.05
0.00
0.003
0.002
0.001
0.000
InvSimpson
Observed
Shannon
Simpson
InvSimpson
Observed
Shannon
Simpson
dif
0.0
−0.1
−0.2
−0.3
0.00
−0.25
−0.50
−0.75
−1.00
0.0
−0.1
−0.2
0.00
−0.05
−0.10
−0.15
InvSimpson
Observed
Shannon
Simpson
