# Supplementary figures and images for: Age-Dependent Gut Microbiota Dynamics and Their Association with Male Life-History Traits in Drosophila melanogaster
Source: Microb Ecol. 2025 Nov 24;89(1):5. doi: 10.1007/s00248-025-02640-y (PMC12743032; doi:10.1007/s00248-025-02640-y)

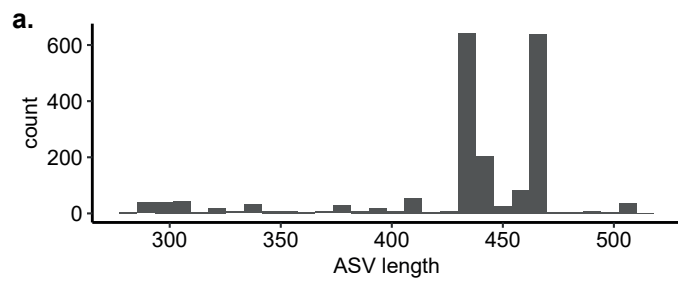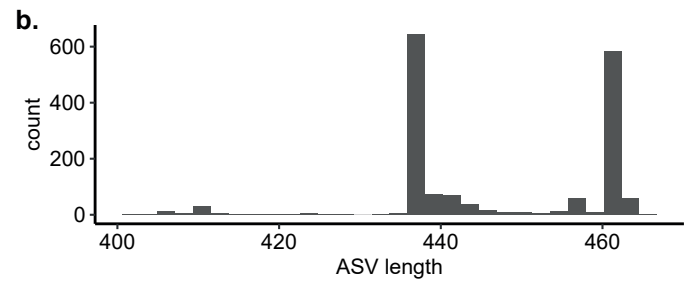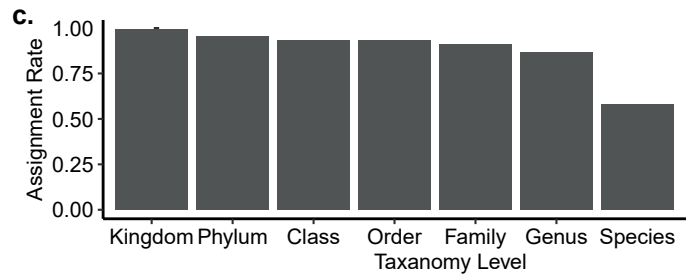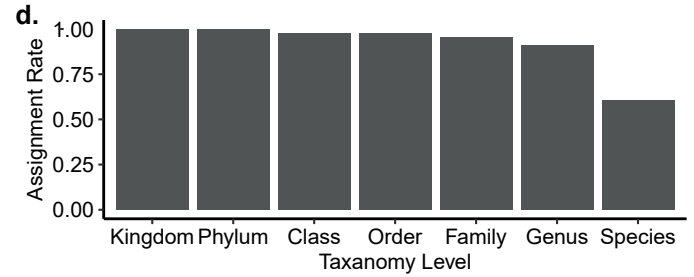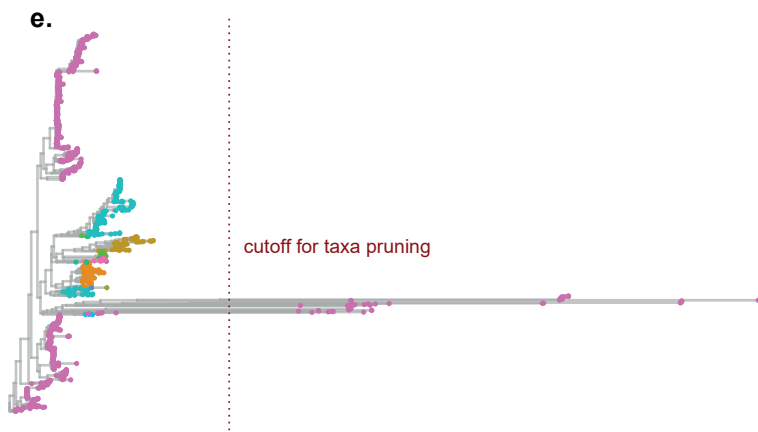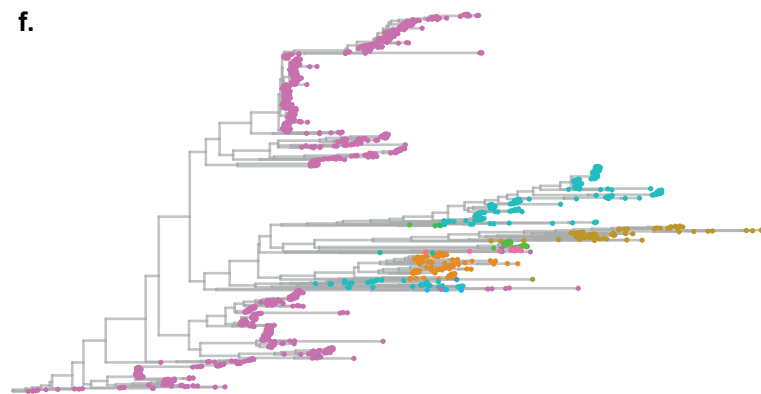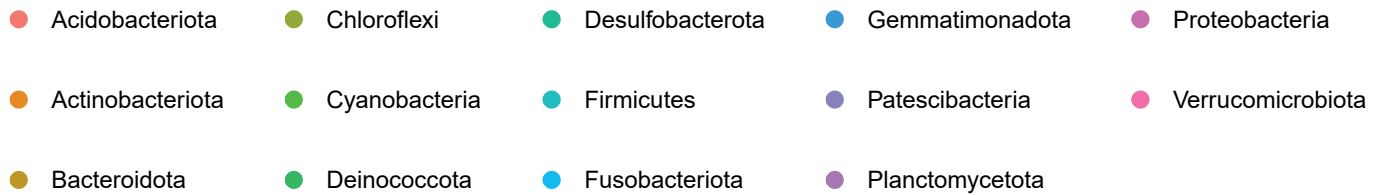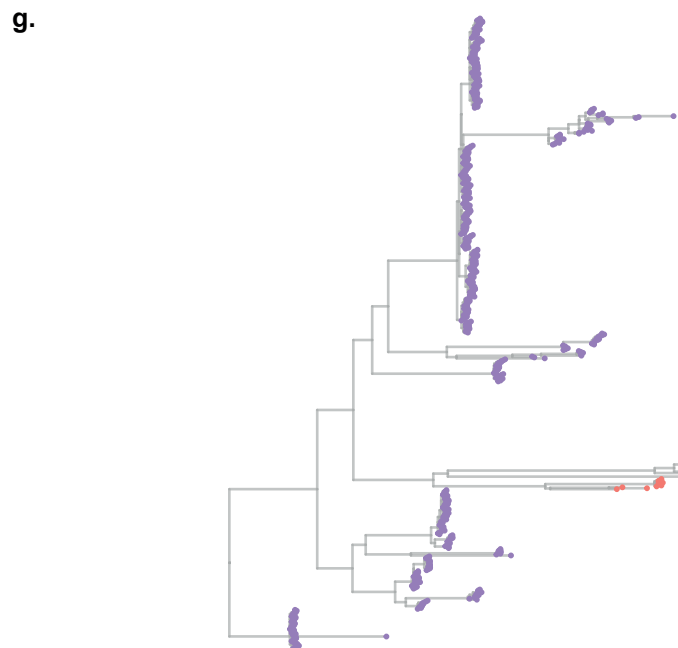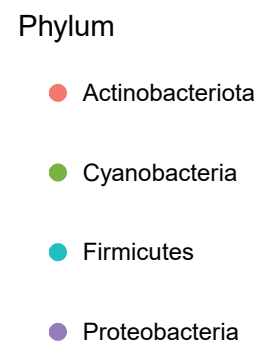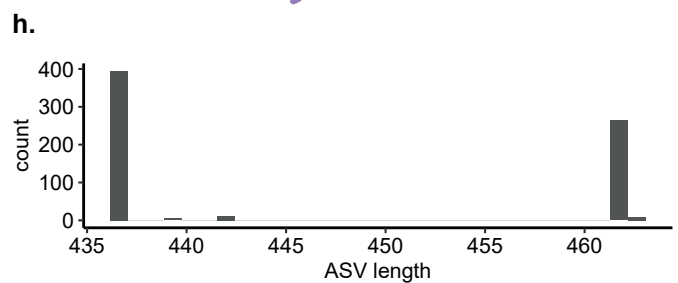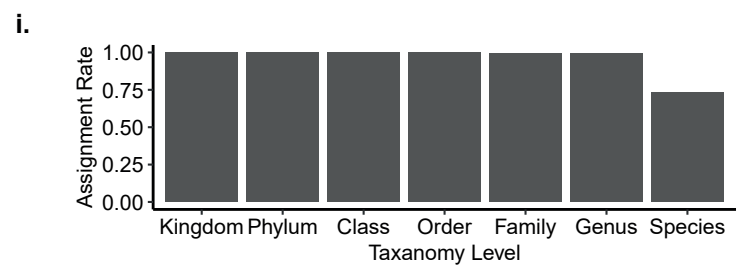

Supplement: Supplementary file 1 — (PDF. 8.56 MB) [file 248_2025_2640_MOESM1_ESM.pdf]

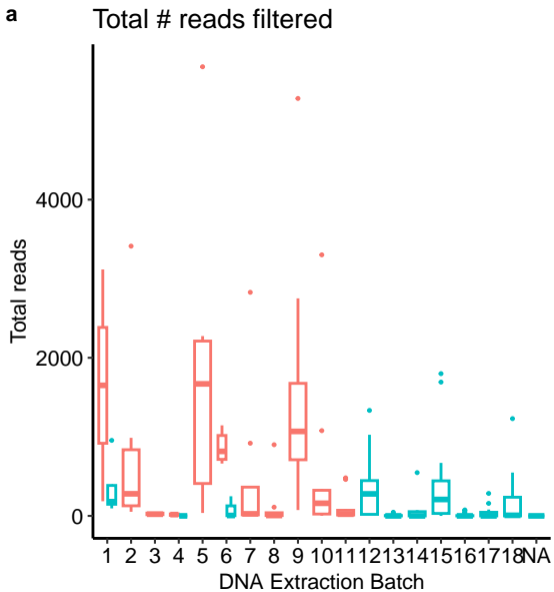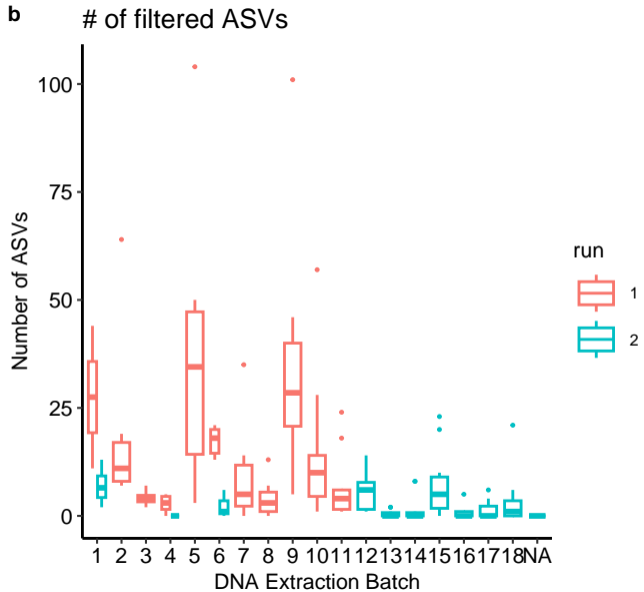

Supplement: Supplementary file 2 — (PDF. 9.83 KB) [file 248_2025_2640_MOESM2_ESM.pdf]

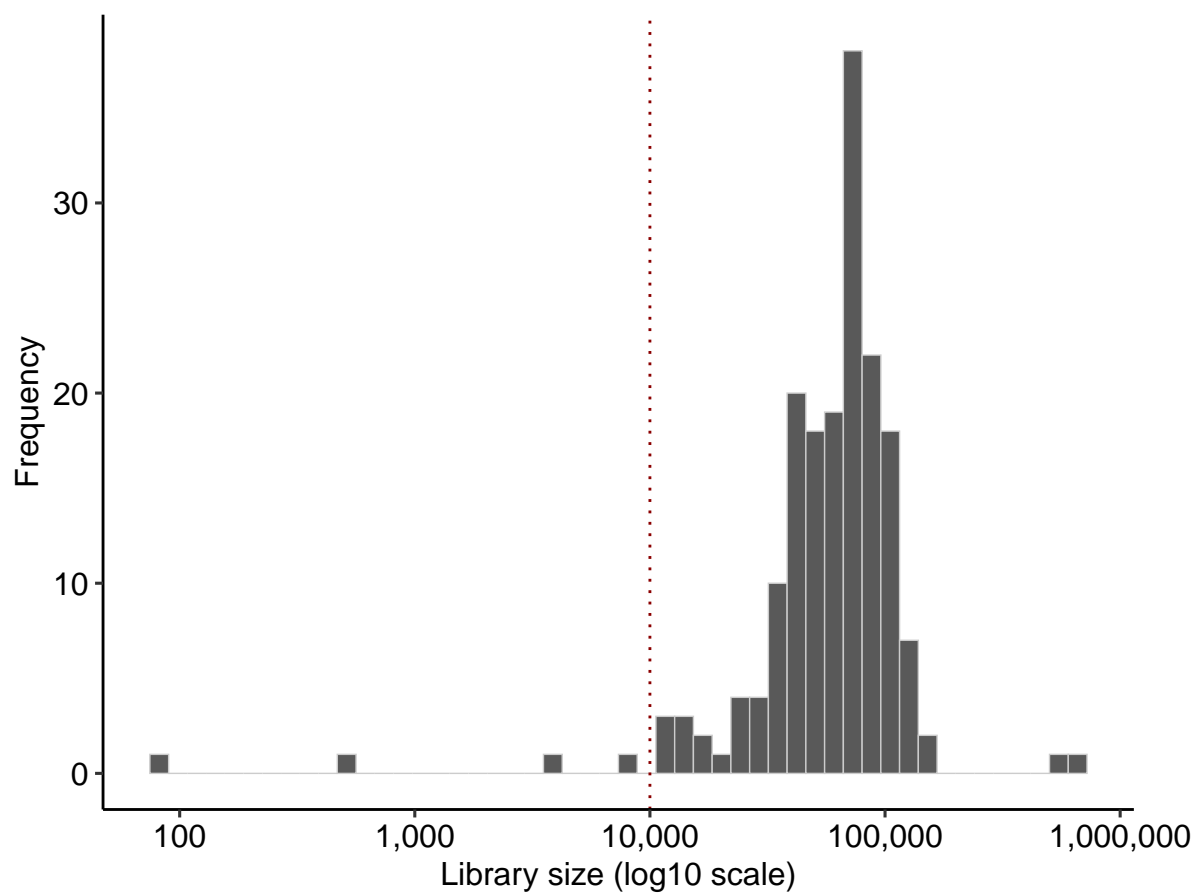

Supplement: Supplementary file 3 — (PDF.4.69 KB) [file 248_2025_2640_MOESM3_ESM.pdf]

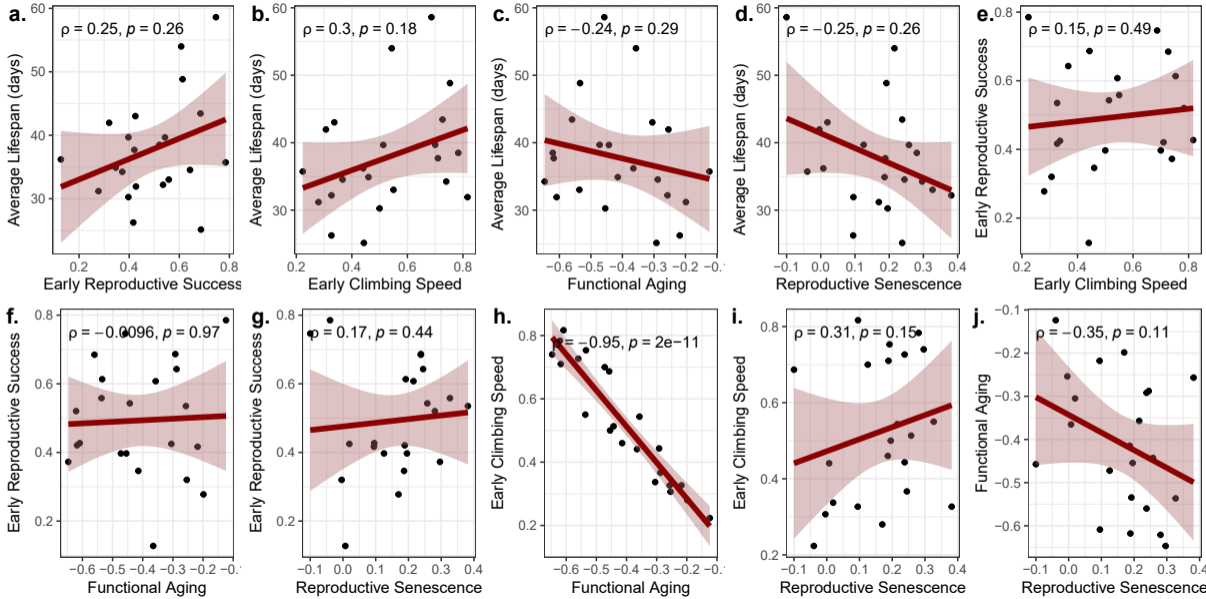

Supplement: Supplementary file 4 — (PDF.352 KB) [file 248_2025_2640_MOESM4_ESM.pdf]

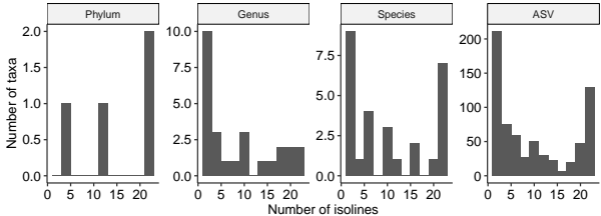

Supplement: Supplementary file 5 — (PDF.5.29 KB) [file 248_2025_2640_MOESM5_ESM.pdf]

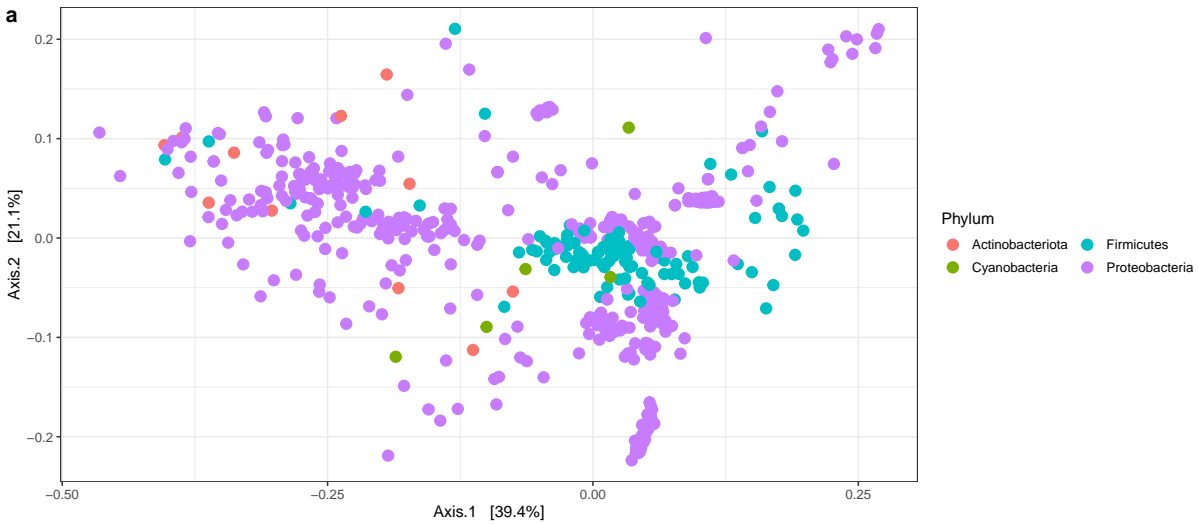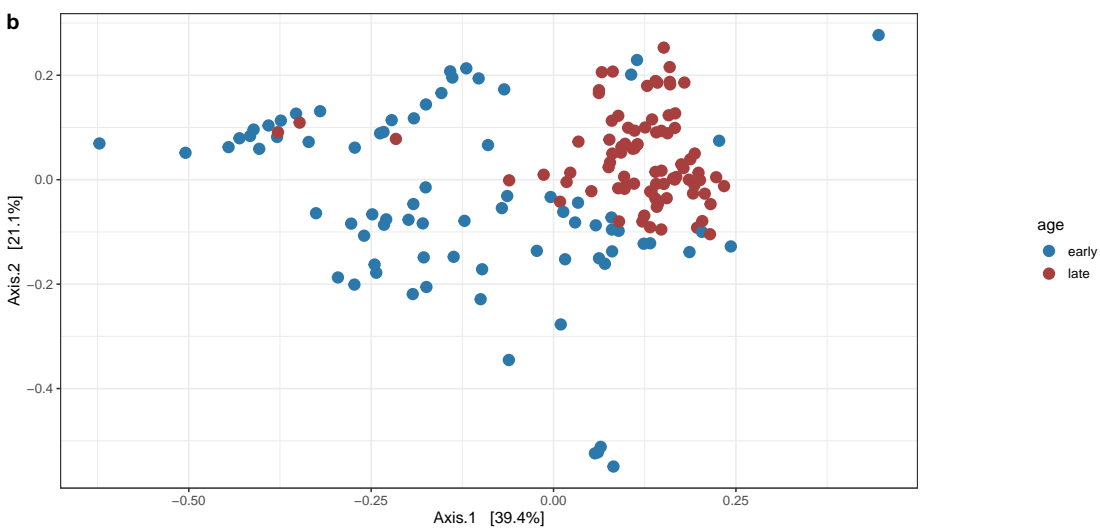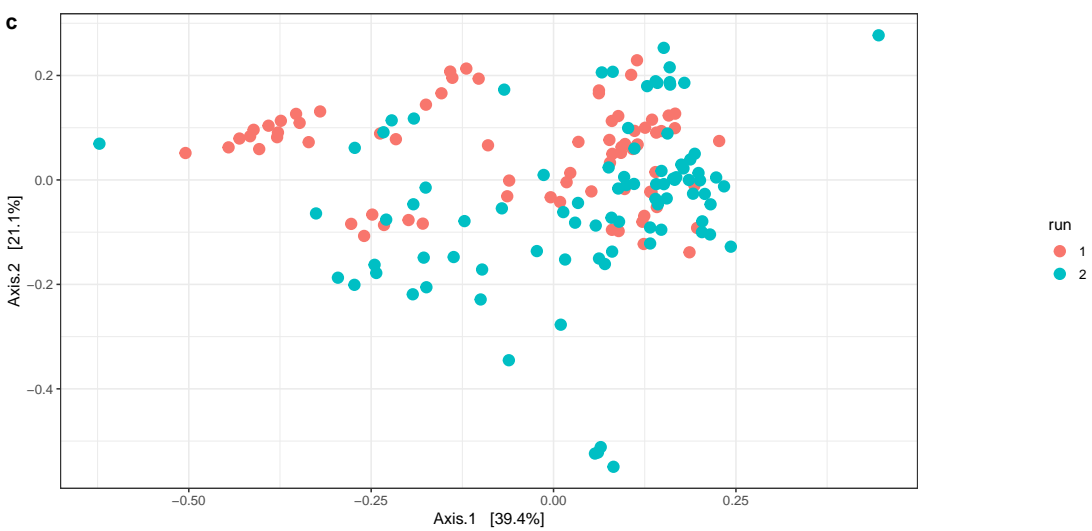

Supplement: Supplementary file 6 — (PDF. 63.4 KB) [file 248_2025_2640_MOESM6_ESM.pdf]

age 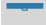 early 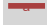 late

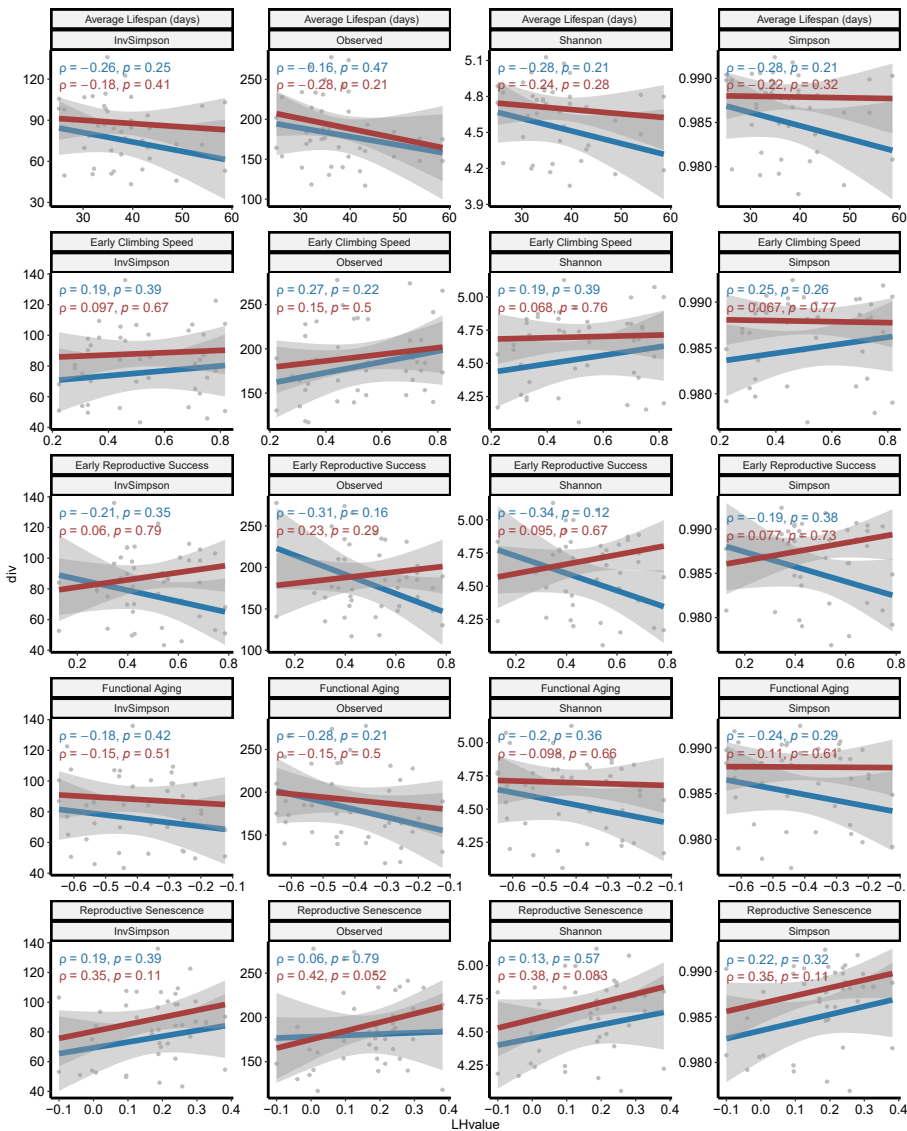

Supplement: Supplementary file 7 — (PDF. 740 KB) [file 248_2025_2640_MOESM7_ESM.pdf]

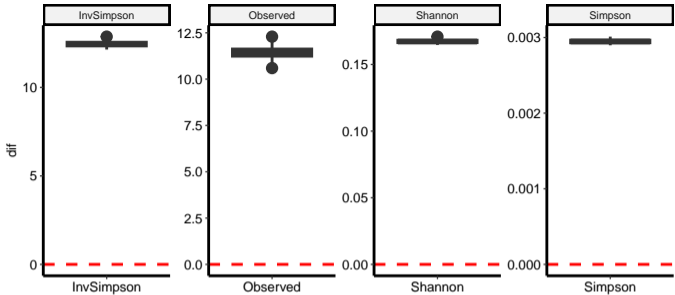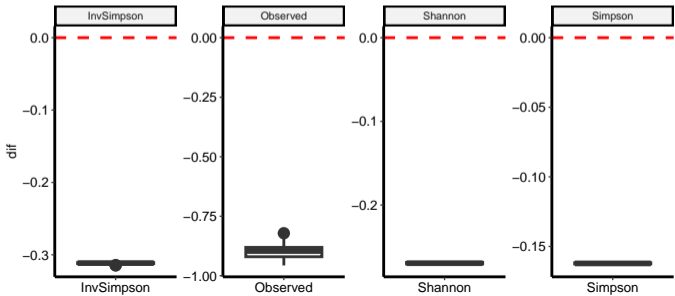

Supplement: Supplementary file 8 — (PDF. 6.35 KB) [file 248_2025_2640_MOESM8_ESM.pdf]
